# Human–animal contact to inform zoonotic disease risk across gradients of agricultural land use change in the Central River Region (CRR) of The Gambia (ZooContact): a formative study

**DOI:** 10.3389/fpubh.2024.1424007

**Published:** 2024-09-10

**Authors:** Aliyu N. Ahmed, Kimberly Fornace, Takuya Iwamura, Kris A. Murray

**Affiliations:** ^1^Centre on Climate Change and Planetary Health, Medical Research Council Unit The Gambia at London School of Hygiene and Tropical Medicine, Fajara, Gambia; ^2^Saw Swee Hock School of Public Health and National University Health Systems, National University of Singapore, Singapore, Singapore; ^3^Institute of Environmental Sciences, University of Geneva, Geneva, Switzerland; ^4^Department of Forest Ecosystems and Society, College of Forestry, Oregon State University, Corvallis, OR, United States

**Keywords:** human animal contact, contact, land-use, zoonosis, risk, agriculture, spillover

## Abstract

**Introduction:**

Pilot studies are important initial steps in research, providing a preliminary assessment of the practicality, feasibility, and potential challenges of a proposed study. This study attempts to assess the feasibility, practicality, and acceptability of a study that integrates a human–animal contact (HAC) questionnaire, animal biodiversity survey using acoustic analysis, and zoonotic disease investigation in animals among rural households in the Central River Region (CRR) of The Gambia. The pilot study revealed granular insights that would otherwise go unnoticed, providing vital information that directly guided the design and implementation of the subsequent full-scale study on zoonotic disease risk.

**Methods:**

A pilot study was conducted in five villages in the CRR of The Gambia. Community sensitization was carried out together with the village leadership, followed by a familiarization tour of the study setting. Questionnaire-based interview was conducted among participants (*n* = 50) randomly selected to assess the acceptability and reliability of the questionnaire. The feasibility and acceptability of biodiversity surveys and animal sampling were assessed using verbal inquiries from participants and community leaders.

**Results:**

The recruitment rate was 96%, and most participants, 50 out of 52, were willing to participate without compensation for lost time during interviews. For animal sampling, 45 out of 50 participants were willing to allow the study team to sample blood and feces from their animals without any form of incentive. All five village heads agreed to the usage of sound recorders to be placed within their community for animal biodiversity assessment. For the survey effort, one field assistant interviewed 25 participants per week. It took a total of 1 h to complete an interview, including random household selection, consenting, and questionnaire interview.

**Discussion:**

The pilot study confirmed the feasibility of the research and informed the design of the larger study. Key parameters, such as community access, acceptability, recruitment success, and logistical requirements, contributed to robust sample size calculations and realistic project cost estimates. Additionally, the study enabled the research team to familiarize themselves with the communities and refine the methods for the full study.

## Introduction

Disentangling the feasibility of a project prior to a full study is crucial to developing and executing a study to obtain reliable outcomes (1, 2). Pilot studies enable the identification of challenges to refine study design and protocol before a full study, allowing for appropriate planning and revision, as well as assessing the suitability of methods for data collection (1–4). Additionally, these studies are useful in exposing research teams to the study communities and helping them fully understand the purpose, method, and procedures of a study in addition to training field staff (3).

Here, we describe a pilot study that was conducted to familiarize a new study team with new study communities and to assess the feasibility and refine the methods of a new project (“ZooContact”). The full project will investigate the role of HAC and local animal biodiversity in affecting pathogen-sharing networks among zoonotic disease hosts and how this relates to zoonotic disease transmission in rural populations of The Gambia, West Africa. ZooContact will comprise a combination of surveys that will run concurrently, involving interviews, animal biodiversity surveys using sound monitors, and animal sampling.

The implementation of such a multidisciplinary project with several parallel surveys could be considerably challenging if not properly planned. The diversity of the methods presents several practical considerations that may constrain the successful implementation of the study. These include inherent challenges with the reliability of the questionnaire, acceptance concerns, difficulty in calculating and achieving robust target sample sizes, obstacles relating to community access, and insufficient engagement with both study personnel and relevant community stakeholders. Therefore, a pilot study to assess the practicability and utility of a draft methodological approach was deemed necessary to clarify challenges and amend the full study protocol for maximum efficacy and cost-effectiveness.

The study uncovered detailed insights on community access, method suitability and acceptability, logistics, and overall feasibility that directly influenced the planning and implementation of the subsequent large-scale investigation.

## Pilot study objectives

The study objectives were to assess the practicability of the study and identify logistic and operational requirements for field activities as follows:

- To familiarize the study team with the communities, project acceptance, and identification of ideal access routes.
- To conduct a pretest of the study questionnaire, evaluating its suitability for quantifying HAC frequency and diversity.
- To assess the willingness of communities to participate in the study and determine whether compensating study participants is necessary.
- To evaluate the acceptability and practicality of taking animal samples and using automated acoustic recorders to conduct animal biodiversity surveys.
- To assess the feasibility of recruiting the predetermined sample size within the study duration and evaluate the required effort and personnel for field activities.

## Methods

### Study area

**Figure 1:**
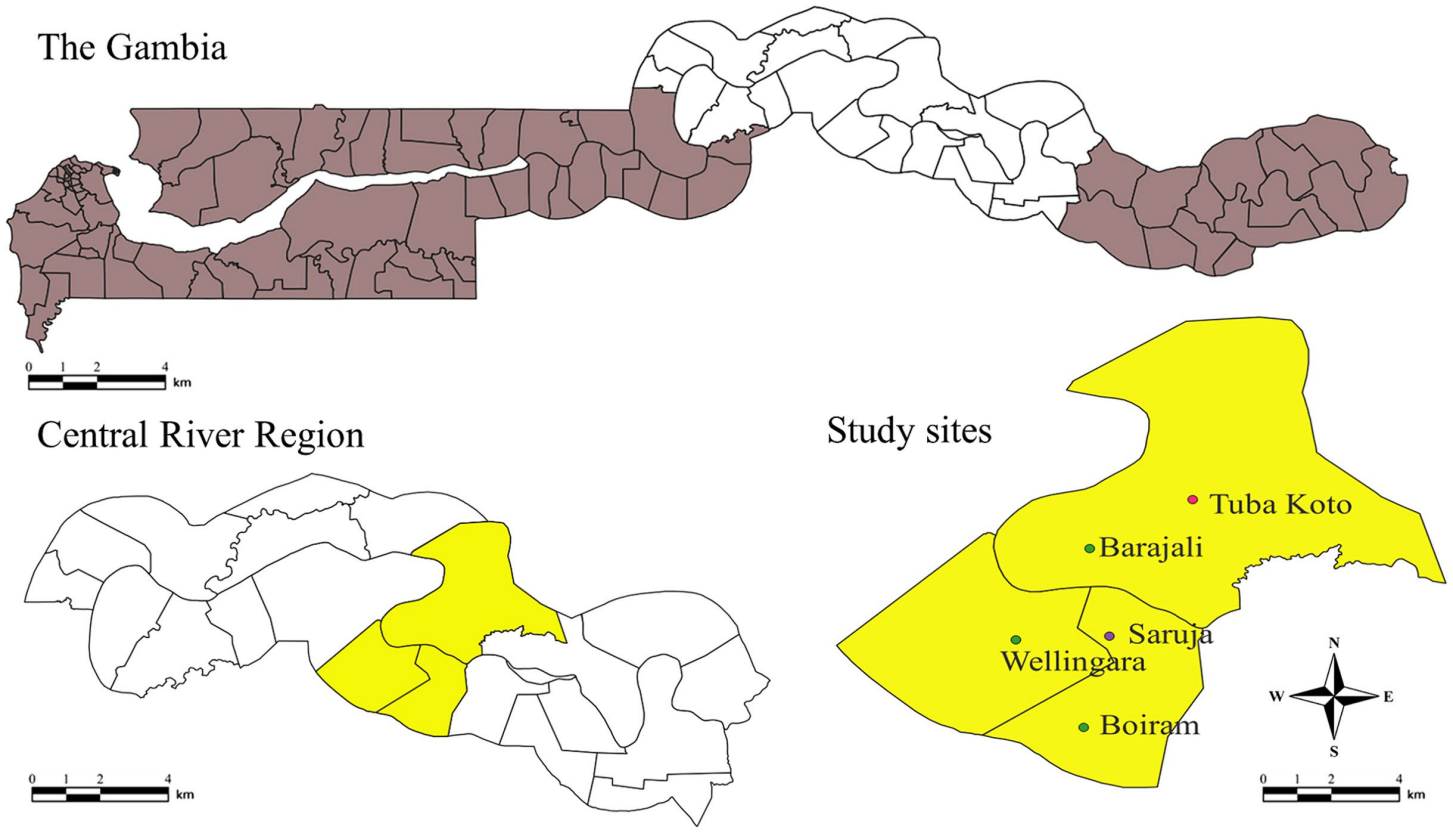
The map of The Gambia showing study sites in CRR.

The study was conducted in the CRR of The Gambia. Five villages were included in the pilot study: Barajally, Tuba Koto, Wellingara, Saruja, and Boiram (Figure 1).

### Study design

The pilot study was designed to align with its objectives, with each aspect of the study carefully considered and implemented to gather relevant data and insights. We used a cross-sectional survey using simple random sampling. Given that the primary aim of the study was to assess the feasibility and refine methods, not to generate data for hypothesis testing, the sample size was not informed by power analysis. We, therefore, adopted a convenience sampling strategy with an *ad hoc* target sample size of 50 households and participants (*n* = 50; 1 per household) across five randomly selected sites based on recommendations from the existing literature (5).

### Recruitment

#### Inclusion criteria

- Male or female aged 15 years and above.
- Residents of the study community.
- Participants who are willing and able to consent to the study.

#### Exclusion criteria

- Individuals aged 15 years and below.
- Non-residents of the pilot study communities.

### Community sensitization

Before the commencement of the study, communities were sensitized through their village head as the facilitator. The study design, purpose, and the study team were introduced to the participants before any activity was conducted.

### Questionnaire development

The questionnaire (Supplementary material S1) was designed using (a) some assessment modules from the Livelihoods and Wellbeing (LivWell) survey tool (6), (b) relevant questions from the Rural Household Multiple Indicator Survey (RHoMIS) (7), (c) similar study questions in literature, and (d) bespoke questions tailored to the study objectives. RHoMIS includes questions focusing on farming practices, livelihoods, and food security, while LivWell focuses on estimating the household livelihood and well-being impacts of environment-related interventions (here, agricultural practices). The survey instrument covers routine household demographics, HAC, agricultural practices, socio-economic livelihood, self-reported health, and wellbeing indicators, as well as knowledge, perceptions, and preventive attitudes toward zoonoses.

### Data collection

A survey of 50 households was used in the study using simple random sampling, with 10 houses selected in each of Wellingara, Saruja, Barajally, Tuba Koto, and Boiram villages. One participant per household was recruited at random to participate in the study after consenting. Before this, each participant was introduced to the study and issued a copy of the study information sheet (Supplementary material S2) to read (or be read to if not literate), understand what the study is about, and decide whether or not to participate. Thereafter, willing participants were issued a consent form (Supplementary material S3) to sign or give their thumbprints as an indication of their voluntary participation.

## Analysis of outcomes

The outcomes for the various objectives were analyzed as follows:

i. *To familiarize the study team with the communities, project acceptance, and identification of ideal access routes*.

Acceptability of the project was assessed using willingness to participate (recruitment rate) and village leadership acceptability (via verbal inquiry) as a proxy for acceptability by the entire community.

Familiarization of the study team was accomplished via a tour around each village. Identifying the best pathway to access the study communities was determined by inquiring directly from the community leaders representing the study villages.

ii. *Pretest and validation of the study questionnaire*.

The acceptability of questionnaires was assessed based on the responses of interviewed participants through verbal inquiry. The duration required to complete an interview was computed as the average time required to complete all participant interviews.

The questionnaire data presented serves the primary purpose of demonstrating the ability of the questionnaire to generate relevant information required for the full study and is not intended for drawing substantive conclusions or results. Subsequent analyses and interpretations are reserved for the full study with a larger and more diverse dataset.

iii. *Willingness to participate and compensation*.

The recruitment rate observed during the study was used as a key indicator of the participants’ willingness to engage and take part in the project. We evaluated the necessity to compensate participants for their time lost during interviews by verbally inquiring about their willingness to participate, irrespective of any remuneration.

iv. *Feasibility of animal sampling and biodiversity survey*.

This was determined by computing the average number of total animal species accessible for sampling in the interviewed compounds and the count of participants expressing positive responses regarding the inclusion of their animals in the subsequent project’s biological sampling.

We determined the feasibility of using automated sound recording for animal biodiversity surveys based on the responses from community leaders “Alkalo” (*n* = 5) to a verbal request to conduct the surveys. Responses were recorded as either “Yes or No.”

v. *Effort required for fieldwork*.

The staff effort required for fieldwork was assessed based on field staff required to complete the study surveys. This was measured from the personnel required to reach the target sample size for the study within the recruitment period of 6 months (projected as fieldwork duration for the full study). It was determined from the number of interviews that could be conducted daily during the survey period by the number of field staff involved. Time effort was estimated from the duration required for all surveys: interviews, biodiversity surveys, and animal sampling to be completed. The commuting effort was assessed based on the total distance covered during the pilot study.

## Result

i. *Familiarization of the study team with the communities, acceptability of the project by the communities, and identification of an ideal community access route*.

**Figure 2:**
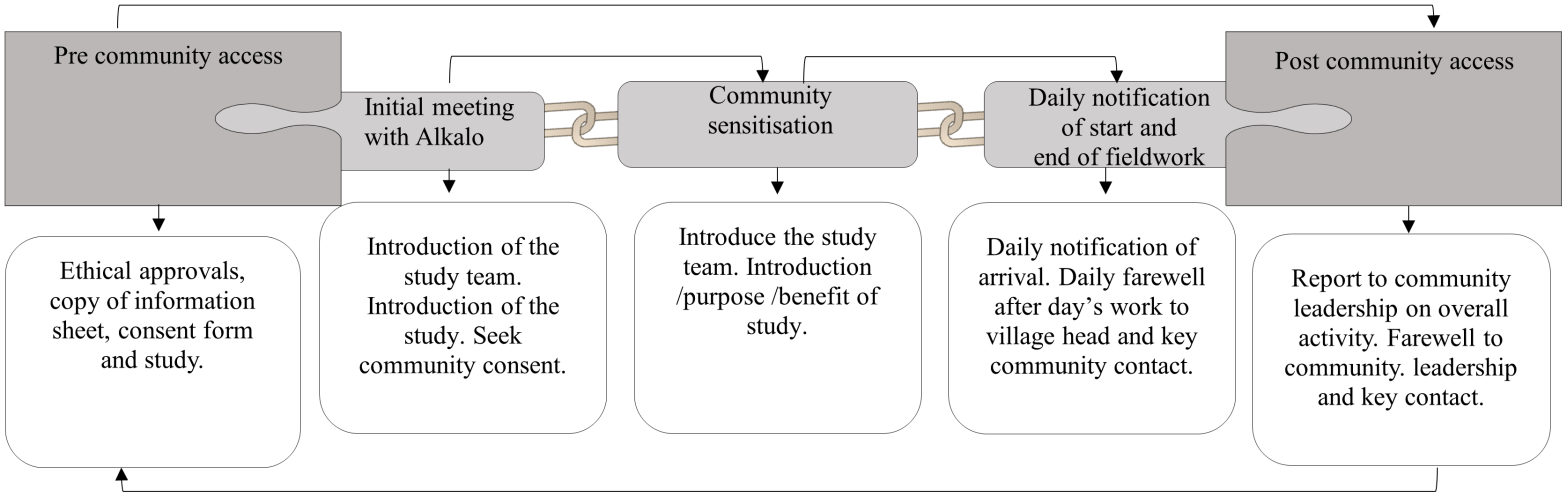
A framework for community access: the figure captures a refined community access plan synthesized from the responses from community leaders. It shows interconnected activities from initial meetings to the notification at the end of the fieldwork, with a detailed description of activities required in boxes below each step from pre- to post-community access.

A framework for access to the study communities (Figure 2) was developed from discussions with the community leaders. All of the village leaders of the five study communities approved of the study pilot. There was a positive response rate of 96% (50 out of 52) from the participants approached for recruitment into the project on behalf of their households.

ii. *Pretest and validation of the study questionnaire*.

For the acceptability of questionnaires, 100% (50 out of 50) of participants reported no concerns. The average time it took to complete a questionnaire was 40 min. It took an average of 10 min for random household selection and another 10 min to recruit and obtain consent from a participant.

**Figure 3:**
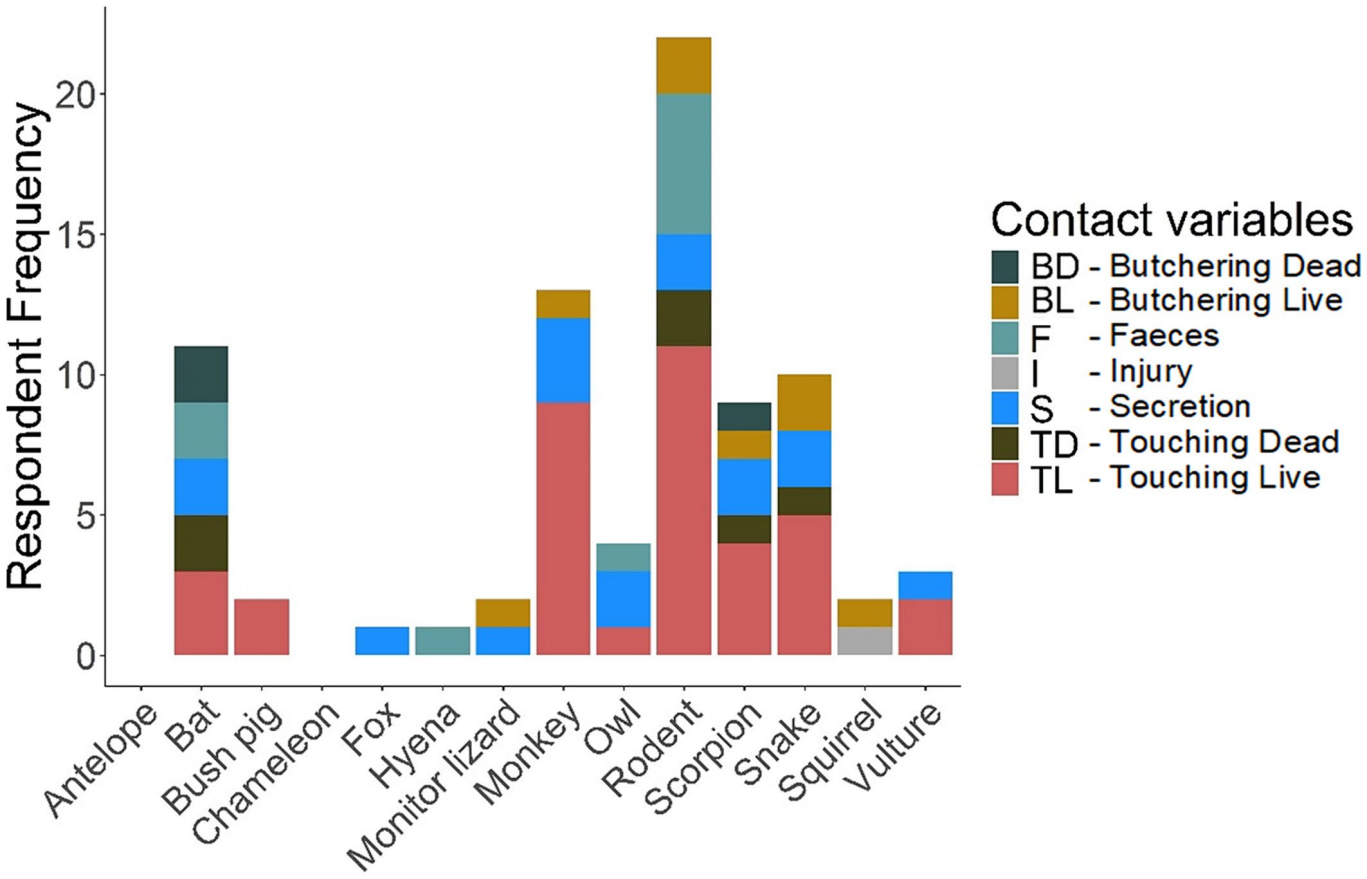
Wild animal contact.

**Figure 4:**
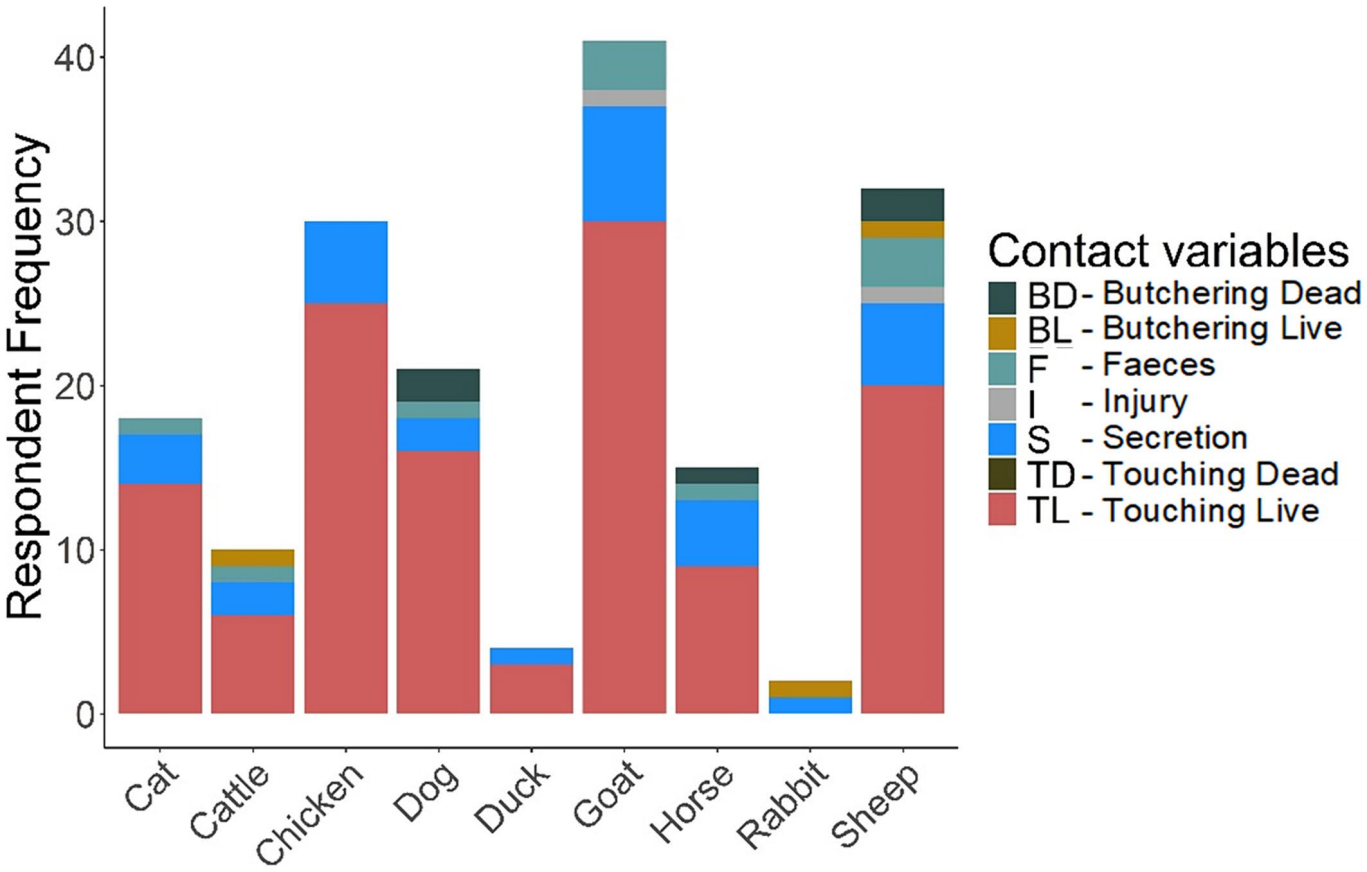
Domestic animal contact.

**Figure 5:**
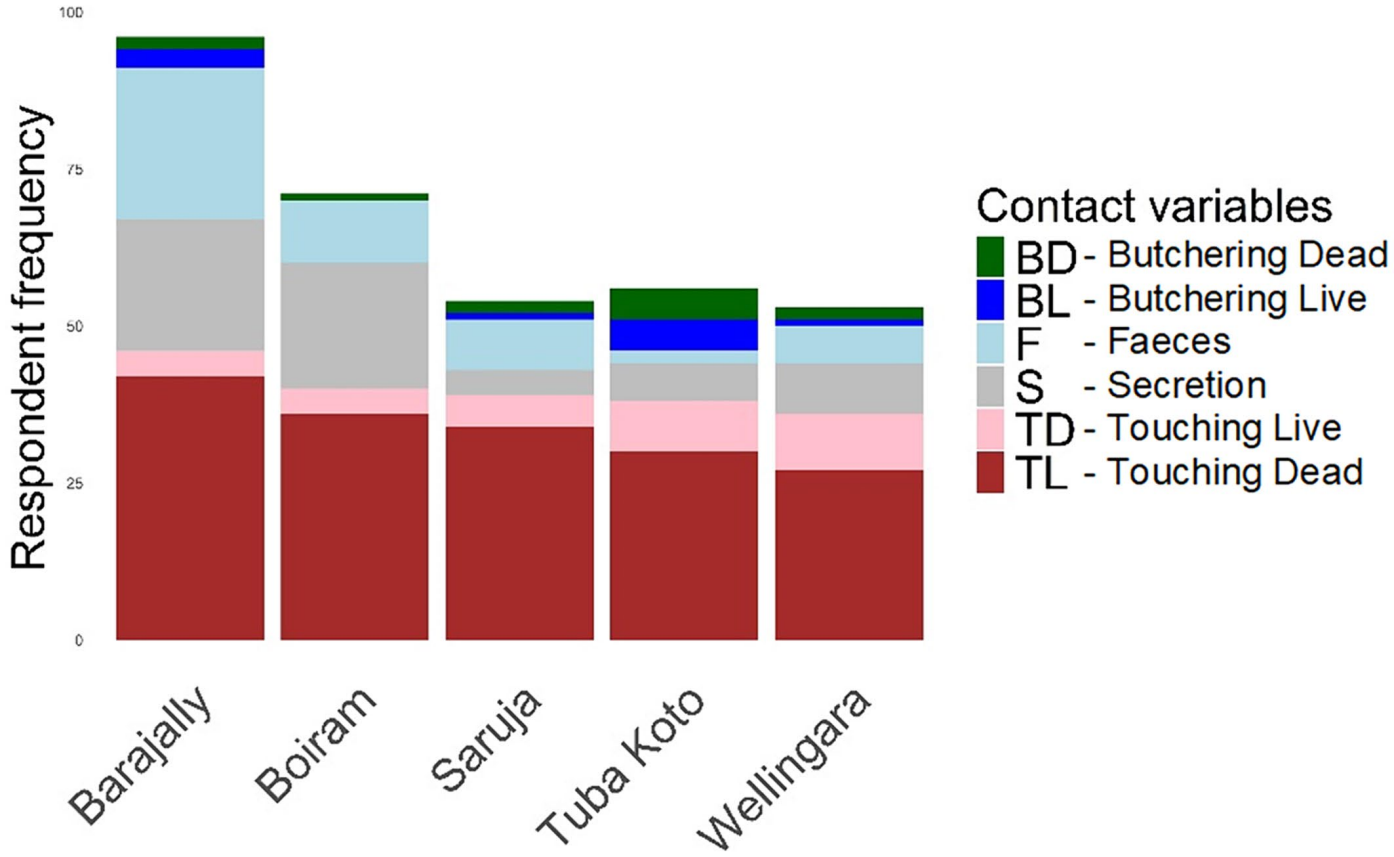
Contact diversity by village.

**Figure 6:**
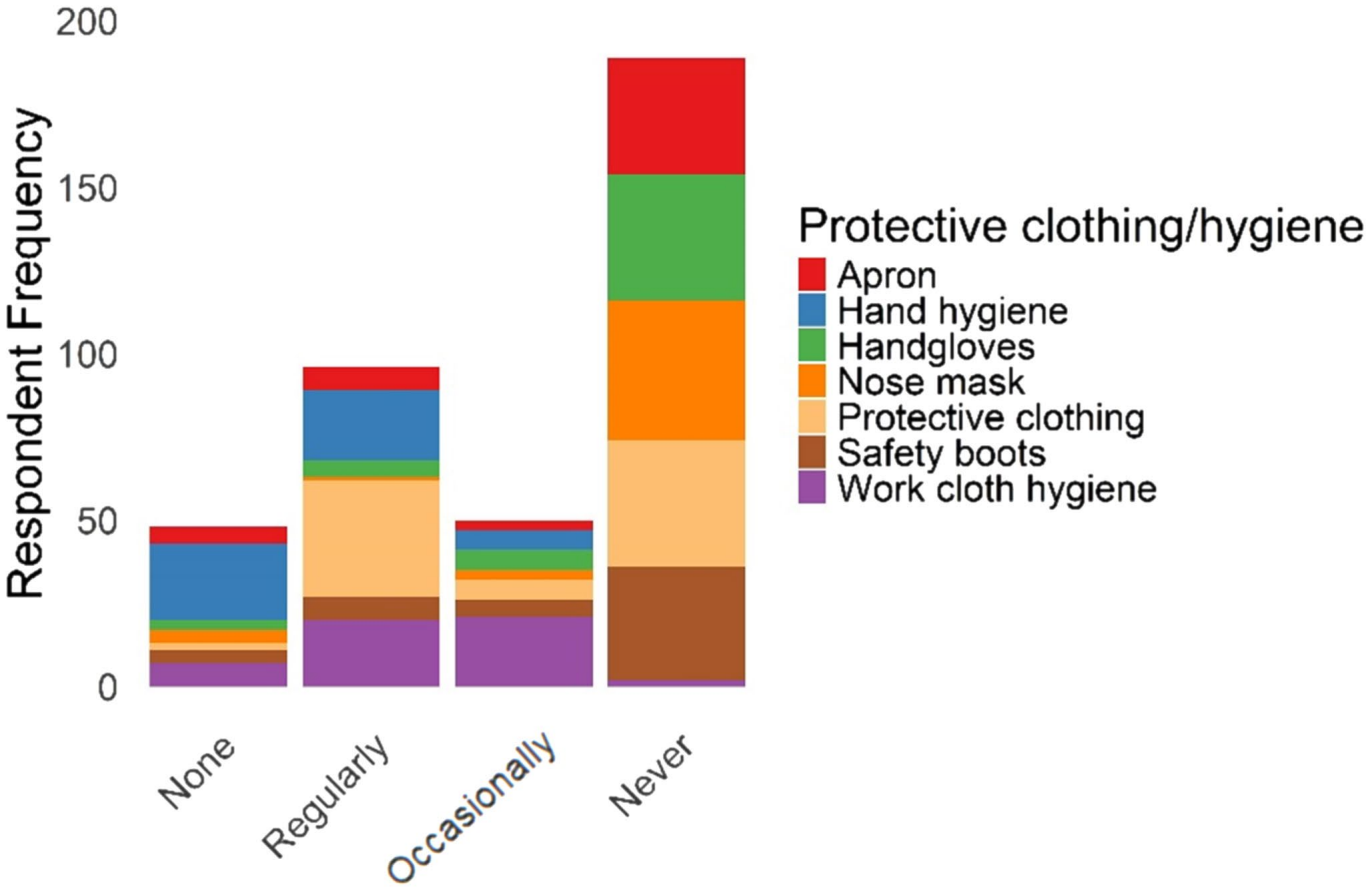
Hygiene practice toward infectious diseases.

The questionnaire results data (Supplementary material S4) generated the required information on the frequency and diversity of animal contacts (see Figures 3–6). More details are provided in Supplementary material S5.

iii. *Willingness to participate and compensation*.

Only 2 out of 52 people approached were not recruited to participate in the study (Table 1), setting the recruitment rate at 96%. The reasons for non-recruitment included those who declined (*n* = 1) and attrition (*n* = 1) (i.e., agreed to participate but were then unavailable for interview).

iv. *Feasibility of animal sampling and animal biodiversity survey*.

**Table 1:** Participants’ willingness to join the study.

		Wellingara	Saruja	Barajally	Tuba Koto	Boiram	Total
Willingness to participate	Questionnaire interview						
	Yes, without incentive	10	10	10	8	8	92% (46/50)
	Yes, with an incentive	0	0	0	1	2	6% (3/50)
	No	0	0	0	1	0	2% (1/50)
	Animal sampling						
	Yes, without incentive	9	10	8	8	10	90% (45/50)
	Yes, with an incentive	1	0	2	1	0	9% (4/50)
	No	0	0	0	1	0	2% (1/50)
	Biodiversity survey	Yes	Yes	Yes	Yes	Yes	100% (5/5)

All participants, 100% (50 out of 50), approved the collection of biological samples from their animals. All village heads (5 out of 5) of the study sites approved using sound recording to quantify local animal biodiversity in their communities.

**Figure 7:**
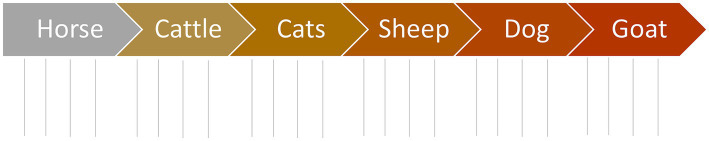
Animal sampling log. The log captures a sampling regimen from the least owned (horses) to the most commonly owned animal species (goats) within the study households. Each animal species sampled will be recorded using a tick mark in the five available line spaces below each species to keep track of the sampling process.

All randomly selected households, 100% (50 out of 50), owned more than one domestic animal species. On average, each household owned (5) animal species, ranging from 1 to 12. This prompted us to consider in more detail an additional study design feature [a bespoke hierarchical sampling technique (Figure 7)] related to ensuring even sampling among species encountered in households, which we developed during the pilot. This method prioritizes sampling from the least common to the most commonly kept animals, to ensure a balanced distribution of sampling across species.

v. *Effort required for fieldwork*.

### Staff and time effort (questionnaire)

It took a total of 1 h to complete one interview, including random house selection (10 min), pleasantries/greetings (10 min), and questionnaire interview (40 min).

### Staff and time effort (animal sampling)

Given that all randomly selected households own more than one animal species, we assumed sampling effort estimates that for each animal, it will take approximately 20 min for three field staff working together to capture, restrain, and sample one (1) domestic animal within a recruited household.

From these data, we developed a mathematical estimation of the total effort required for the full study (Table 2).

**Table 2:** Time and effort required to complete the proposed fieldwork for the full study.

Parameter	Formula	Values
Staff and time effort (questionnaire)		
Total time for one interview (*Tti*)	*Tti* = *Tp* + *THH* + *Ti*	*Tti* = 10 min + 10 min + 40 min = 60 min per interview
Number of total interviews per day (*NtiPd*)	$NtiPd=\frac{Nw}{Tti}$	$NtiPd=\frac{5h}{Tti}$ × 60 min = 5 interviews per day
Number of total interviews per week (*NtiPW*)	*NtiPW* = *NtiPd* × *Nw*	*NtiPW* = 5 interviews per day × 5 days = 25 interviews per week
Total time for interviews per day (*TtiPD*)	*TtiPD* = *NtiPd* × *Tti*	*TtiPD* = 5 interviews per day × 60 min = 300 min per day (5 h)
Total time for interviews per week (*TtiPW*)	*TtiPW* = *NtiPW* × *Tti*	*TtiPW* = 25 interviews per week × 60 min = 1,500 min (25 h)
Effort for one week of fieldwork (*Effort one week*)	*Effort one week* = *Tk* + *K* × *TtiPW*	*Effort one week* = *Tk* + *K* × *TtiPW* = 1 h + 1.2 × 1,500 min = 1 h + 30 h = 31 h
Number of field workers required (*Nfieldworkers*)	*Nfieldwrokers* = $\frac{Nti}{NtiPW\times Ntw}$	$=\frac{792}{25\times 16}=1.98=$ 2 field assistants
Staff and time effort (animal sampling)		
	*T* total = *T*per × *n*	*T* total = 20 min/animal × 792 animals = 15,840 min (264 h)

Definition of terms: *Tk* = Take off time, *Tp* = Time for pleasantries, *THH* = Time for random HH selection, *Ti* = Time for interview, *Tti* = Time for total interview, *Tw* = Working hours per day, *TtiPD* = Time for total interviews per day, *TtiPW* = Time for total interviews per week, *Nti* = Number of total target interviews for full study, *Nw* = Number of working days per week, *Ntw* = Total weeks available for field work, *NtiPd* = Number of total interviews per day, *NtiPW* = Number of total interviews per week, *K* = buffer accounting for uncertainties, and *Effort one week* = Effort for 1 week of fieldwork by one interviewer. Given values: *Tk* = 1 h, *THH* = 10 min, *Tp* = 10 min, *Ti* = 40 min, *Tw* = 5 h, *Nw* = 5 days, *Nti* = 792 participants, *Ntw* = 16 weeks, and *K* (buffer accounting for uncertainties) = 1.2 (20%). Definition of terms: *T* per animal = time taken to sample one animal, *n* = number of animals. Given values: *T* per animal = 20 min, *n* = number of animals is 792. *T* total = 20 min/animal × 792 animals. *T* total = 15,840 min (264 h).

Hence, 1 h and 20 min are required for both the questionnaire interview and animal sampling per household, which will be run in parallel by two separate teams of two and three field assistants working together to achieve a target sample size within 6 months available for field activities.

### Commuting effort

**Figure 8:**
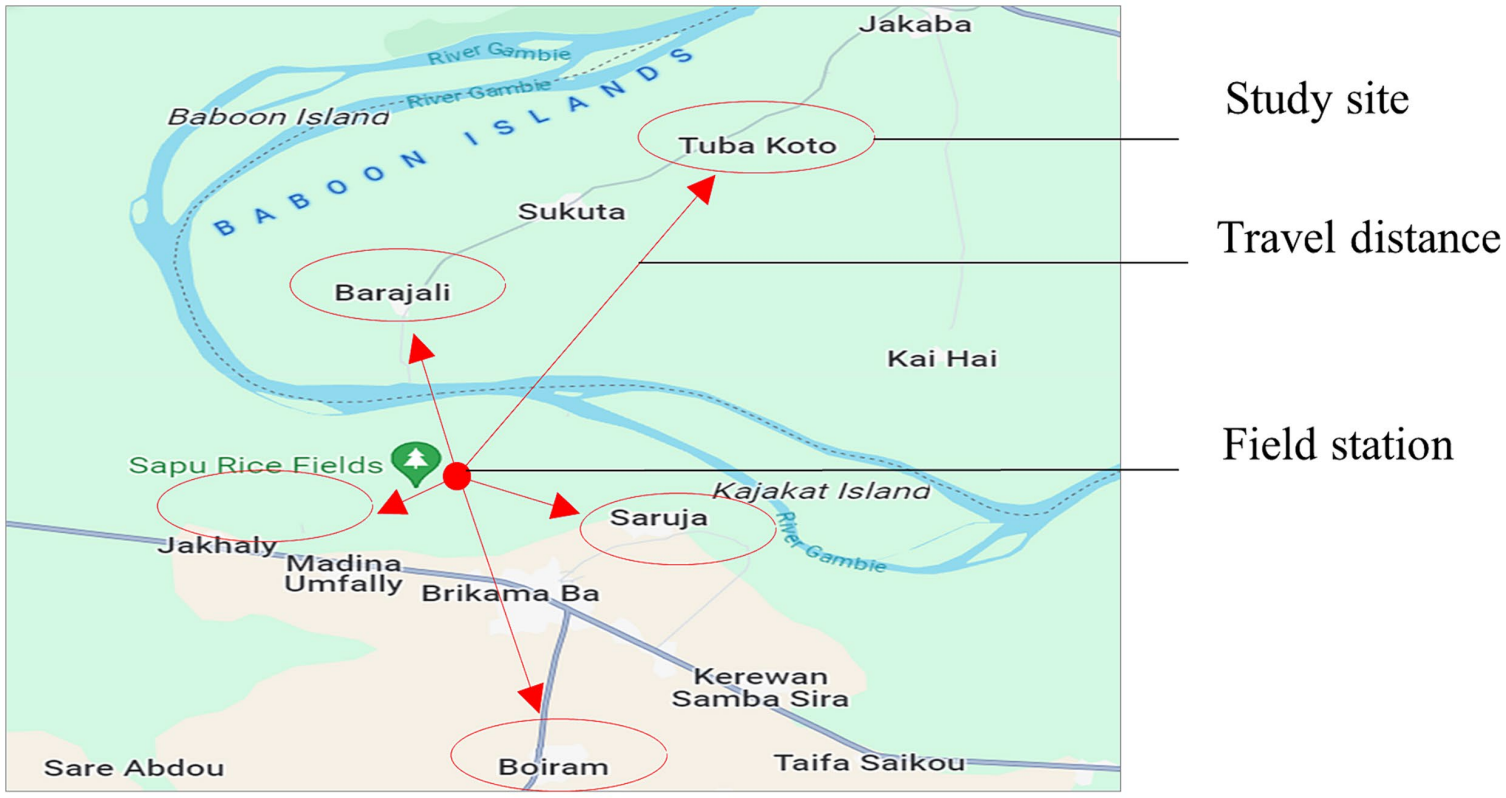
The Google map shows the estimation of the distance traveled from the field station to the study sites.

We assessed the total travel distance covered by the approximate distance between pilot study sites and the field station (Table 3) on Google Maps to estimate travel effort (Figure 8). This guided us in designing the transport logistics for the larger study (Table 4).

**Table 3:** Estimated travel time and distance to pilot study communities.

S/N	Travel to the field site	Estimated distance (km)	Estimated transit time (min)
1	Walikunda to Barajally Suba	3.80 km × 8 trips	20 min × 8 trips
2	Walikunda to Saruja	3.70 km × 8 trips	8 min × 8 trips
3	Walikunda to Boiram	12.20 km × 8 trips	27 min × 8 trips
4	Walikunda to Wellingara	2.10 km × 8 trips	4 min × 8 trips
5	Walikunda to Tuba Koto	8.90 km × 8 trips	12 min × 8 trips
6	Fajara to Walikunda	277 km × 4 (two return trips)	272 min × 4 (two return trips)
7	Miscellaneous trips	50 km	60 min
Total		1402.60 km	1716.00 min
Average		233.7 km	286 min

**Table 4:** Estimated travel distance for the proposed full study.

Parameter	Formula	Values
Travel distance		
Average travel distance (*A*) for one village		$A=\frac{80+3.70+12.20+2.10+8.90}{5}=\frac{30.70}{5}$ = 6.14 km per village
Travel distance (*T*) for 12 villages using average distance	*T* = *A* × *n*	*T* = 6.14 × 12 = 6.14 × 12 = 73.68 km
Total travel distance (*T* estimate) for 12 villages using constant distance (*Kd*)	*T* estimate = *T* + *K*	*T* estimate = 73.68 + 327 = 400.68 km
Travel time		
Average time (*At*) for one village		*At* = 20 × 8 + 8 × 8 + 27 × 8 + 4 × 8 + 12 × 8 ÷ 5 = 177.6 min per village
Time (*Tt*) for 12 villages using average time	*Tt* = *A* × *n*	*Tt* = 6.14 × 12 = 6.14 × 12 = 73.68 km
Total travel time (*T* estimate) for 12 villages using constant distance (*Kt*)	*Tt* estimate = *T* + *Kt*	*Tt* estimate = 177.6 × 12 = 2131.2 min

Definition of terms: *A* = Average travel distance, *T* = Travel distance for all study sites, *At* = Average travel time, *Tt* = Travel time for all study sites, *n* = Number of study sites, *Kd* = Distance of travel to field station and miscellaneous, and *Kt* = Time of travel to the field station and miscellaneous. Given values: *A* = 6.14 km, *At* = 177.6 min, *Kd* = 277 × 4 = 50 = 1,158 km, and *Kt* = 272 × 4 = 50 = 1,138 km.

## Discussion

The study demonstrated that conducting “ZooContact” was feasible. The study provided valuable insights that informed the redesign and revision of the full-scale study. Initially, the study team introduced themselves to the village heads and sought their approval to conduct the study within their communities. After receiving approval, the community leaders disseminated the information to the entire community through social centers, including prayer grounds, community centers, and market stalls, facilitating community preparation for the study. Thereafter, the team took a tour around the community to familiarize themselves with the village setting. Two field assistants served as an intermediary between the study team and the communities to facilitate the process. The use of these assistants, who are familiar with the communities, immediately bred a sense of trust in the study team.

We consulted with the village leaders to determine the most suitable approach for the study team’s access to their communities from the start to the end of the study. All village heads recommended an initial meeting with them as the community leaders, after which they would inform the entire community. The recruitment process for participants included an introductory briefing about the project and then providing them with a more detailed information sheet. Those voluntarily willing to participate were then asked to give informed consent before proceeding. This level of engagement is critical for ensuring robust engagement and reliable data.

The questionnaire was positively received, with all participants, 100% (50 out of 50), expressing no concerns and indicating that no compensation for time lost during the interviews was necessary. A total of 25 participants were recruited per week, with 5 interviewed daily on average. We recorded the start and end times of all interviews and estimated the average time it takes to complete a questionnaire to be 40 min. The average completion time of 40 min for the questionnaires is comparable to other studies, such as Peters et al. (8), who noted that rural health assessments typically take between 30 and 45 min. This similarity suggests that the questionnaire design was both user-friendly and efficient, contributing to the overall feasibility of the study. A notable recruitment rate of 96% (50 out of 52) shows strong community willingness to participate, with minimal attrition (2 out of 52). This high level of engagement supports findings from other community-based studies. For instance, formative research on community-based agriculture-to-nutrition trial in rural Malawi highlighted the crucial role of early community engagement and pilot testing in achieving high participation rates (9). The study demonstrated that addressing cultural and logistical factors, engaging local leaders, and using recommended access routes are effective strategies for achieving high participation rates. This method aligns with Carpenter’s et al. (10) findings, which also emphasized the importance of local leadership in maintaining high levels of participant engagement in community-based research.

There was unanimous approval from community leaders (5 out of 5) for an acoustic animal biodiversity survey, and suitable locations for positioning sound monitors were identified. Furthermore, 100% approval was recorded for animal biological sample collection from all study communities, reflecting strong community support. Similar results are corroborated by Yadana et al. (11), who reported a 98% approval rate in a livestock disease surveillance project, highlighting the importance of thorough community engagement in epidemiological research. We estimated approximately 20 min for three (3) field personnel to capture, restrain, and sample one (1) domestic animal within a household. The estimate was made crudely, predicated upon the feasibility metrics that all houses interviewed owned an average of five (5) species of animals, with most households having their animal housing within their compound, and the majority of these animals were used to handling, which makes it easier for the field team to sample. This aligns with observations and findings by Carpenter et al. (10), who reported 22 min per animal sampling, further reinforcing the practicality of our time estimates.

Although the pilot study revealed that the average household owned multiple domestic animals, there was an unequal distribution of animal species available. This finding necessitated the design of a hierarchical sampling technique to ensure even sampling among targeted species. This method will ensure systematic sampling and minimize bias and errors, as highlighted by Drewe et al. (12) in their study, where they highlighted quantitative techniques to optimize sampling strategies, ensuring that the data collected from various livestock populations were representative and reliable.

The overall interview process, including house selection, initial interactions, and the questionnaire, was completed in approximately 1 h. This efficiency highlights the feasibility of scaling up the study for larger implementations through a streamlined approach that can be effectively scaled without compromising data quality. The same approach was adopted by the van Klink et al. (13) study, where they successfully optimized their data collection methods by implementing a systematic technique that reduced time and resource requirements, achieving streamlined processes that allowed for efficient large-scale data collection.

The staff effort estimation, given the comprehensive nature of the field activities, required an estimated 1 h and 20 min per household for both the questionnaire interview and animal sampling. These activities would be conducted in parallel by two separate teams: one team of two (2) field assistants would handle the interviews, while another team of three (3) would manage animal sampling. This approach is designed to ensure efficiency and to attain the target sample size within the 6 months allocated for field activities in the full study. The task distribution and its parallel execution are critical for maintaining efficiency and meeting the study’s timelines.

### Limitations of the study

Although the study revealed several strengths, such as high participation rates and efficient data collection processes, potential limitations are acknowledged. The sample size of 50 participants, while adequate for a pilot, may limit the generalizability of the findings to larger populations. For instance, the time required for random household selection and participant recruitment could vary widely depending on community size, accessibility, and household distribution. Anticipating these logistical issues is crucial to planning the full study.

Additionally, the uniformly high acceptance rates might reflect implicit selection bias, where more cooperative communities were involved in the pilot. The larger study therefore anticipates a more diverse range of community acceptance and considers this in the study design. Furthermore, the reliance on self-reported data for the acceptability of questionnaires and willingness to participate can be subject to social desirability bias, where respondents may provide answers they perceive to be favorable rather than their true opinions. Larson (14) discusses how social desirability and memory biases can affect the accuracy of self-reported data, highlighting the need for careful consideration of these factors in interpreting results. Furthermore, the pilot study did not account for seasonal variations, which could affect animal availability, participant recruitment rate, and community engagement (15). Therefore, including a broader temporal scope in future studies could provide more comprehensive insights.

## Conclusion

Overall, this pilot study demonstrated the feasibility of conducting the “ZooContact” study. The findings provided valuable insights that informed the full study protocol, including community access strategies, recruitment processes, and logistical requirements. The robust participation rates and streamlined data collection processes observed in the pilot study suggest that scaling up to the larger study is feasible. Integrating these insights into the full study design will maximize efficiency, community engagement, and data integrity, ensuring successful implementation.

Decisions that informed the final protocol include the following:

- The framework for community access (Figure 2) provides a structured approach to guide access to each study community in the full study.
- Conducting interviews and animal sampling concurrently has proven feasible.
- The animal sampling log (Figure 7) will serve as a guide for the animal sampling process.
- The required sampling effort indicates that the full study will necessitate five field personnel: two for conducting interviews and three for carrying out animal sampling, working in parallel to achieve the target sample size within the 6-month fieldwork period.
- Compensation for participants who lost working hours was not necessary for the full study.

## Data Availability

The original contributions presented in the study are included in the article/Supplementary material, further inquiries can be directed to the corresponding author.
