# Supplementary material for: Human–animal contact to inform zoonotic disease risk across gradients of agricultural land use change in the Central River Region (CRR) of The Gambia (ZooContact): a formative study
Source: Front Public Health. 2024 Sep 10;12:1424007. doi: 10.3389/fpubh.2024.1424007 (PMC11419968; doi:10.3389/fpubh.2024.1424007)
Supplement: Supplementary file 1 [file Table_1.DOCX]

**
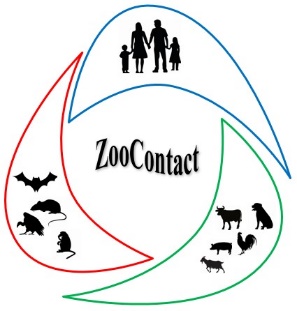
**

**ZooContact Questionnaire**

| Cover page | | |
| --- | --- | --- |
| Questionnaire ID | Town / Village / House / Serial No. |  |
| Interview date | DD / MM / MM |  |
| Interview time | HH: / MM AM PM |  |
| Interviewer name |  |  |
| Household GPS coordinate |  |  |
| Elevation |  |  |
| Participant serial no |  |  |

| A. Socio-demographic information | | | | | | | | | | | | | | | | | | | | | | | | | | | | | | | | | | | | | | | |
| --- | --- | --- | --- | --- | --- | --- | --- | --- | --- | --- | --- | --- | --- | --- | --- | --- | --- | --- | --- | --- | --- | --- | --- | --- | --- | --- | --- | --- | --- | --- | --- | --- | --- | --- | --- | --- | --- | --- | --- |
|  |  |  | | | | | | | | | | | | | | | | | | | | | | | | | | | | | | | | | |  |  | | |
|  | Age |  | | | | | | | | | | | | | | | | | | | | | | | | | | | | | | | | | | |  | | |
|  | Sex | Male | | | | | | | | | | | | | | | | | | | | | | | | | | | | | | | | | | 0 |  | | |
|  |  | Female | | | | | | | | | | | | | | | | | | | | | | | | | | | | | | | | | | 1 |  |  |  |
|  | Marital status | Single | | | | | | | | | | | | | | | | | | | | | | | | | | | | | | | | | | 1 |  | | |
|  |  | Married | | | | | | | | | | | | | | | | | | | | | | | | | | | | | | | | | | 2 |  |  |  |
|  |  | Divorced | | | | | | | | | | | | | | | | | | | | | | | | | | | | | | | | | | 3 |  |  |  |
|  |  | Widowed | | | | | | | | | | | | | | | | | | | | | | | | | | | | | | | | | |  |  |  |  |
|  | Education | Primary education | | | | | | | | | | | | | | | | | | | | | | | | | | | | | | | | | | 1 |  | | |
|  |  | Secondary education | | | | | | | | | | | | | | | | | | | | | | | | | | | | | | | | | | 2 |  |  |  |
|  |  | Tertiary education | | | | | | | | | | | | | | | | | | | | | | | | | | | | | | | | | | 3 |  |  |  |
|  |  | Informal | | | | | | | | | | | | | | | | | | | | | | | | | | | | | | | | | | 4 |  |  |  |
|  |  | Not educated | | | | | | | | | | | | | | | | | | | | | | | | | | | | | | | | | | 5 |  |  |  |
|  | Primary occupation | Farmer | | | | | | | | | | | | | | | | | | | | | | | | | | | | | | | | | | 1 |  | | |
|  |  | Herdsman/animal husbandry | | | | | | | | | | | | | | | | | | | | | | | | | | | | | | | | | | 2 |  |  |  |
|  |  | Fisherman | | | | | | | | | | | | | | | | | | | | | | | | | | | | | | | | | | 3 |  |  |  |
|  |  | Forest related work | | | | | | | | | | | | | | | | | | | | | | | | | | | | | | | | | | 4 |  |  |  |
|  |  | Trader | | | | | | | | | | | | | | | | | | | | | | | | | | | | | | | | | | 5 |  |  |  |
|  |  | Labourer | | | | | | | | | | | | | | | | | | | | | | | | | | | | | | | | | | 6 |  |  |  |
|  |  | Hunter | | | | | | | | | | | | | | | | | | | | | | | | | | | | | | | | | | 7 |  |  |  |
|  |  | Civil servant | | | | | | | | | | | | | | | | | | | | | | | | | | | | | | | | | | 8 |  |  |  |
|  |  | Housewife | | | | | | | | | | | | | | | | | | | | | | | | | | | | | | | | | | 9 |  |  |  |
|  |  | Student | | | | | | | | | | | | | | | | | | | | | | | | | | | | | | | | | | 10 |  |  |  |
|  |  | Retired | | | | | | | | | | | | | | | | | | | | | | | | | | | | | | | | | | 11 |  |  |  |
|  |  | Unemployed | | | | | | | | | | | | | | | | | | | | | | | | | | | | | | | | | | 12 |  |  |  |
|  |  | Other | | | | | | | | | | | | | | | | | | | | | | | | | | | | | | | | | | 13 |  |  |  |
|  | Years on work |  | | | | | | | | | | | | | | | | | | | | | | | | | | | | | | | | | | |  | | |
|  | Cultural group | Mandinka | | | | | | | | | | | | | | | | | | | | | | | | | | | | | | | | | | 1 |  | | |
|  |  | Wolof | | | | | | | | | | | | | | | | | | | | | | | | | | | | | | | | | | 2 |  |  |  |
|  |  | Fula | | | | | | | | | | | | | | | | | | | | | | | | | | | | | | | | | | 3 |  |  |  |
|  |  | Jola | | | | | | | | | | | | | | | | | | | | | | | | | | | | | | | | | | 4 |  |  |  |
|  |  | Other | | | | | | | | | | | | | | | | | | | | | | | | | | | | | | | | | | 5 |  |  |  |
|  |  |  | | | | | | | | | | | | | | | | | | | | | | | | | | | | | | | | | |  |  |  |  |
|  | Years of occupancy in community |  | | | | | | | | | | | | | | | | | | | | | | | | | | | | | | | | | | |  | | |
|  | Children | Male | | | | | | | | | | | | | | | | | | | | | | | | | | | | | | | | | |  |  | | |
|  |  | Female | | | | | | | | | | | | | | | | | | | | | | | | | | | | | | | | | |  |  |  |  |
| B. Household information | | | | | | | | | | | | | | | | | | | | | | | | | | | | | | | | | | | | | | | |
| Household occupant details | | | | | | | | | | | | | | | | | | | | | | | | | | | | | | | | | | | | | | | |
|  | Number of occupants | Total occupants | | | | | | | | | | | | | | | | | | | | | | | | | | | | | | | | | | |  | | |
|  |  | Male occupants | | | | | | | | | | | | | | | | | | | | | | | | | | | | | | | | | | |  |  |  |
|  |  | Female occupants | | | | | | | | | | | | | | | | | | | | | | | | | | | | | | | | | | |  |  |  |
|  |  | Children (below 18) | | | | | | | | | | | | | | | | | | | | | | | | | | | | | | | | | | |  |  |  |
|  | Employment status | Formal employment | | | | | | | | | | | | | | | | | | | | | | | | | | | | | | | | | | |  | | |
|  |  | Informal employment | | | | | | | | | | | | | | | | | | | | | | | | | | | | | | | | | | |  |  |  |
|  |  | Unemployed | | | | | | | | | | | | | | | | | | | | | | | | | | | | | | | | | | |  |  |  |
|  | Education status | Formal education | | | | | | | | | | | | | | | | | | | | | | | | | | | | | | | | | | |  | | |
|  |  | Informal education | | | | | | | | | | | | | | | | | | | | | | | | | | | | | | | | | | |  |  |  |
|  |  | Uneducated | | | | | | | | | | | | | | | | | | | | | | | | | | | | | | | | | | |  |  |  |
| Vectors  (Perceived household vectors) | | | | | | | | | | | | | | | | | | | | | | | | | | | | | | | | | | | | | | | |
|  | Mosquitoes | High | | | | | | | | | | | | | | | | | | | | | | | | | | | | | | | | | | 1 |  | | |
|  |  | Low | | | | | | | | | | | | | | | | | | | | | | | | | | | | | | | | | | 2 |  |  |  |
|  |  | Medium | | | | | | | | | | | | | | | | | | | | | | | | | | | | | | | | | | 3 |  |  |  |
|  | Flies | High | | | | | | | | | | | | | | | | | | | | | | | | | | | | | | | | | | 1 |  | | |
|  |  | Low | | | | | | | | | | | | | | | | | | | | | | | | | | | | | | | | | | 2 |  |  |  |
|  |  | Medium | | | | | | | | | | | | | | | | | | | | | | | | | | | | | | | | | | 3 |  |  |  |
|  | Lice | High | | | | | | | | | | | | | | | | | | | | | | | | | | | | | | | | | | 1 |  | | |
|  |  | Low | | | | | | | | | | | | | | | | | | | | | | | | | | | | | | | | | | 2 |  |  |  |
|  |  | Medium | | | | | | | | | | | | | | | | | | | | | | | | | | | | | | | | | | 3 |  |  |  |
|  | Fleas | High | | | | | | | | | | | | | | | | | | | | | | | | | | | | | | | | | | 1 |  | | |
|  |  | Low | | | | | | | | | | | | | | | | | | | | | | | | | | | | | | | | | | 2 |  |  |  |
|  |  | Medium | | | | | | | | | | | | | | | | | | | | | | | | | | | | | | | | | | 3 |  |  |  |
|  | Ticks | High | | | | | | | | | | | | | | | | | | | | | | | | | | | | | | | | | | 1 |  | | |
|  |  | Low | | | | | | | | | | | | | | | | | | | | | | | | | | | | | | | | | | 2 |  |  |  |
|  |  | Medium | | | | | | | | | | | | | | | | | | | | | | | | | | | | | | | | | | 3 |  |  |  |
|  | Bugs | High | | | | | | | | | | | | | | | | | | | | | | | | | | | | | | | | | | 1 |  | | |
|  |  | Low | | | | | | | | | | | | | | | | | | | | | | | | | | | | | | | | | | 2 |  |  |  |
|  |  | Medium | | | | | | | | | | | | | | | | | | | | | | | | | | | | | | | | | | 3 |  |  |  |
| Animals within and around household  Past 1 month | | | | | | | | | | | | | | | | | | | | | | | | | | | | | | | | | | | | | | | |
|  | Pets  (list numbers) | Dogs | | | | | | | | | | | | | | | | | | | | | | | | | | | | | | | | | | |  | | |
|  |  | Cats | | | | | | | | | | | | | | | | | | | | | | | | | | | | | | | | | | |  |  |  |
|  | Livestock  (list numbers) | Chicken | | | | | | | | | | | | | | | | | | | | | | | | | | | | | | | | | | |  | | |
|  |  | Duck | | | | | | | | | | | | | | | | | | | | | | | | | | | | | | | | | | |  |  |  |
|  |  | Rabbit | | | | | | | | | | | | | | | | | | | | | | | | | | | | | | | | | | |  |  |  |
|  |  | Sheep | | | | | | | | | | | | | | | | | | | | | | | | | | | | | | | | | | |  |  |  |
|  |  | Goat | | | | | | | | | | | | | | | | | | | | | | | | | | | | | | | | | | |  |  |  |
|  |  | Cattle | | | | | | | | | | | | | | | | | | | | | | | | | | | | | | | | | | |  |  |  |
|  |  | Donkey | | | | | | | | | | | | | | | | | | | | | | | | | | | | | | | | | | |  |  |  |
|  |  | Horses | | | | | | | | | | | | | | | | | | | | | | | | | | | | | | | | | | |  |  |  |
|  |  |  | | | | | | | | | | | | | | | | | | | | | | | | | | | | | | | | | | |  |  |  |
|  | Wildlife (Seen within or around the household) | Monkey | | | | | | | | | | | | | | | | | | | | | | | | | | | | | | | | | | 1 |  | | |
|  |  | Snakes | | | | | | | | | | | | | | | | | | | | | | | | | | | | | | | | | | 2 |  |  |  |
|  |  | Hyena | | | | | | | | | | | | | | | | | | | | | | | | | | | | | | | | | | 3 |  |  |  |
|  |  | Squirrel | | | | | | | | | | | | | | | | | | | | | | | | | | | | | | | | | | 4 |  |  |  |
|  |  | Bush pigs | | | | | | | | | | | | | | | | | | | | | | | | | | | | | | | | | | 5 |  |  |  |
|  |  | Fox | | | | | | | | | | | | | | | | | | | | | | | | | | | | | | | | | | 6 |  |  |  |
|  |  | Rodents | | | | | | | | | | | | | | | | | | | | | | | | | | | | | | | | | | 7 |  |  |  |
|  |  | Rabbit | | | | | | | | | | | | | | | | | | | | | | | | | | | | | | | | | | 8 |  |  |  |
|  |  | Antelope | | | | | | | | | | | | | | | | | | | | | | | | | | | | | | | | | | 9 |  |  |  |
|  |  | Scorpion | | | | | | | | | | | | | | | | | | | | | | | | | | | | | | | | | | 10 |  |  |  |
|  |  | Monitor lizard | | | | | | | | | | | | | | | | | | | | | | | | | | | | | | | | | | 11 |  |  |  |
|  |  | Chameleon | | | | | | | | | | | | | | | | | | | | | | | | | | | | | | | | | | 12 |  |  |  |
|  |  | Owl | | | | | | | | | | | | | | | | | | | | | | | | | | | | | | | | | | 13 |  |  |  |
|  |  | Bats | | | | | | | | | | | | | | | | | | | | | | | | | | | | | | | | | | 14 |  |  |  |
|  |  | Wild birds | | | | | | | | | | | | | | | | | | | | | | | | | | | | | | | | | | 15 |  |  |  |
|  |  | Others _____________________________________________ | | | | | | | | | | | | | | | | | | | | | | | | | | | | | | | | | | 16 |  |  |  |
| Vegetation within household | | | | | | | | | | | | | | | | | | | | | | | | | | | | | | | | | | | | | | | |
|  | Fruit trees | Mangoes | | | | | | | | | | | | | | | | | | | | | | | | | | | | | | | | | | 1 |  | | |
|  |  | Banana | | | | | | | | | | | | | | | | | | | | | | | | | | | | | | | | | | 2 |  |  |  |
|  |  | Cashew | | | | | | | | | | | | | | | | | | | | | | | | | | | | | | | | | | 3 |  |  |  |
|  |  | Orange | | | | | | | | | | | | | | | | | | | | | | | | | | | | | | | | | | 4 |  |  |  |
|  |  | Pawpaw | | | | | | | | | | | | | | | | | | | | | | | | | | | | | | | | | | 5 |  |  |  |
|  |  | Baobab | | | | | | | | | | | | | | | | | | | | | | | | | | | | | | | | | | 6 |  |  |  |
|  |  | Guava | | | | | | | | | | | | | | | | | | | | | | | | | | | | | | | | | | 8 |  |  |  |
|  |  | Grape | | | | | | | | | | | | | | | | | | | | | | | | | | | | | | | | | | 9 |  |  |  |
|  |  | Other | | | | | | | | | | | | | | | | | | | | | | | | | | | | | | | | | | 10 |  |  |  |
|  | Plant trees | Neem | | | | | | | | | | | | | | | | | | | | | | | | | | | | | | | | | | 1 |  |  |  |
|  |  | Doctor plant | | | | | | | | | | | | | | | | | | | | | | | | | | | | | | | | | | 2 |  |  |  |
|  |  | Moringa | | | | | | | | | | | | | | | | | | | | | | | | | | | | | | | | | | 3 |  |  |  |
|  |  | Malena | | | | | | | | | | | | | | | | | | | | | | | | | | | | | | | | | | 5 |  |  |  |
|  |  | Mahogany | | | | | | | | | | | | | | | | | | | | | | | | | | | | | | | | | | 6 |  |  |  |
|  |  | Date tree | | | | | | | | | | | | | | | | | | | | | | | | | | | | | | | | | | 10 |  |  |  |
|  |  | Other______________________________________ | | | | | | | | | | | | | | | | | | | | | | | | | | | | | | | | | |  |  |  |  |
|  | Shrubs |  | | | | | | | | | | | | | | | | | | | | | | | | | | | | | | | | | |  |  |  |  |
|  | Crops  (Past 12 months) | ___________________________________________ | | | | | | | | | | | | | | | | | | | | | | | | | | | | | | | | | | |  |  |  |
| Household utilities | | | | | | | | | | | | | | | | | | | | | | | | | | | | | | | | | | | | | | | |
|  | Electricity | Main Grid | | | | | | | | | Primary | | | | | | | | | | | | | | | | | | | | | | | | | 1 |  | | |
|  |  |  |  |  |  |  |  |  |  |  | Secondary | | | | | | | | | | | | | | | | | | | | | | | | | 2 |  |  |  |
|  |  | Solar | | | | | | | | | Primary | | | | | | | | | | | | | | | | | | | | | | | | | 1 |  |  |  |
|  |  |  |  |  |  |  |  |  |  |  | Secondary | | | | | | | | | | | | | | | | | | | | | | | | | 2 |  |  |  |
|  |  | Generator | | | | | | | | | Primary | | | | | | | | | | | | | | | | | | | | | | | | | 1 |  |  |  |
|  |  |  |  |  |  |  |  |  |  |  | Secondary | | | | | | | | | | | | | | | | | | | | | | | | | 2 |  |  |  |
|  | Water supply | Piped from main supply | | | | | | | | | Primary | | | | | | | | | | | | | | | | | | | | | | | | | 1 |  |  |  |
|  |  |  |  |  |  |  |  |  |  |  | Secondary | | | | | | | | | | | | | | | | | | | | | | | | | 2 |  |  |  |
|  |  | Well in compound | | | | | | | | | Primary | | | | | | | | | | | | | | | | | | | | | | | | | 1 |  |  |  |
|  |  |  |  |  |  |  |  |  |  |  | Secondary | | | | | | | | | | | | | | | | | | | | | | | | | 2 |  |  |  |
|  |  | Well within the community | | | | | | | | | Primary | | | | | | | | | | | | | | | | | | | | | | | | | 1 |  |  |  |
|  |  |  |  |  |  |  |  |  |  |  | Secondary | | | | | | | | | | | | | | | | | | | | | | | | | 2 |  |  |  |
|  |  | Stream/river | | | | | | | | | Primary | | | | | | | | | | | | | | | | | | | | | | | | | 1 |  |  |  |
|  |  |  |  |  |  |  |  |  |  |  | Secondary | | | | | | | | | | | | | | | | | | | | | | | | | 2 |  |  |  |
|  | Water distribution | Piped | | | | | | | | | Primary | | | | | | | | | | | | | | | | | | | | | | | | | 1 |  |  |  |
|  |  |  |  |  |  |  |  |  |  |  | Secondary | | | | | | | | | | | | | | | | | | | | | | | | | 2 |  |  |  |
|  |  | Hose | | | | | | | | | Primary | | | | | | | | | | | | | | | | | | | | | | | | | 1 |  |  |  |
|  |  |  |  |  |  |  |  |  |  |  | Secondary | | | | | | | | | | | | | | | | | | | | | | | | | 2 |  |  |  |
|  |  | Manual hauling | | | | | | | | | Primary | | | | | | | | | | | | | | | | | | | | | | | | | 1 |  |  |  |
|  |  |  |  |  |  |  |  |  |  |  | Secondary | | | | | | | | | | | | | | | | | | | | | | | | | 2 |  |  |  |
|  | Fuel source | Firewood | | | | | | | | | Primary | | | | | | | | | | | | | | | | | | | | | | | | | 1 |  |  |  |
|  |  |  |  |  |  |  |  |  |  |  | Secondary | | | | | | | | | | | | | | | | | | | | | | | | | 2 |  |  |  |
|  |  | Charcoal | | | | | | | | | Primary | | | | | | | | | | | | | | | | | | | | | | | | | 1 |  |  |  |
|  |  |  |  |  |  |  |  |  |  |  | Secondary | | | | | | | | | | | | | | | | | | | | | | | | | 2 |  |  |  |
|  |  | Gas | | | | | | | | | Primary | | | | | | | | | | | | | | | | | | | | | | | | | 1 |  |  |  |
|  |  |  |  |  |  |  |  |  |  |  | Secondary | | | | | | | | | | | | | | | | | | | | | | | | | 2 |  |  |  |
|  |  | Electric | | | | | | | | | Primary | | | | | | | | | | | | | | | | | | | | | | | | | 1 |  |  |  |
|  |  |  |  |  |  |  |  |  |  |  | Secondary | | | | | | | | | | | | | | | | | | | | | | | | | 2 |  |  |  |
|  |  | Kerosene | | | | | | | | | Primary | | | | | | | | | | | | | | | | | | | | | | | | | 1 |  |  |  |
|  |  |  |  |  |  |  |  |  |  |  | Secondary | | | | | | | | | | | | | | | | | | | | | | | | | 2 |  |  |  |
|  | Toilet system | Pit latrine | | | | | | | | | Primary | | | | | | | | | | | | | | | | | | | | | | | | | 1 |  |  |  |
|  |  |  |  |  |  |  |  |  |  |  | Secondary | | | | | | | | | | | | | | | | | | | | | | | | | 2 |  |  |  |
|  |  | Flush toilet | | | | | | | | | Primary | | | | | | | | | | | | | | | | | | | | | | | | | 1 |  |  |  |
|  |  |  |  |  |  |  |  |  |  |  | Secondary | | | | | | | | | | | | | | | | | | | | | | | | | 2 |  |  |  |
|  |  | Open field or bush | | | | | | | | | Primary | | | | | | | | | | | | | | | | | | | | | | | | | 1 |  |  |  |
|  |  |  |  |  |  |  |  |  |  |  | Secondary | | | | | | | | | | | | | | | | | | | | | | | | | 2 |  |  |  |
| C. Human-animal contact | | | | | | | | | | | | | | | | | | | | | | | | | | | | | | | | | | | | | | | |
| Contact moments (have you come in contact within the past 3 month during these activities) | | | | | | | | | | | | | | | | | | | | | | | | | | | | | | | | | | | | | | | |
|  | Occupational | Hunting | | | | | | | | | | | | | | | | | | | | | | | | | | | | | | | | | | 1 |  | | |
|  |  | Slaughter | | | | | | | | | | | | | | | | | | | | | | | | | | | | | | | | | | 2 |  |  |  |
|  |  | Wildlife attack | | | | | | | | | | | | | | | | | | | | | | | | | | | | | | | | | | 3 |  |  |  |
|  |  | Wildlife trade | | | | | | | | | | | | | | | | | | | | | | | | | | | | | | | | | | 4 |  |  |  |
|  |  | Farming | | | | | | | | | | | | | | | | | | | | | | | | | | | | | | | | | | 5 |  |  |  |
|  |  | Fishing | | | | | | | | | | | | | | | | | | | | | | | | | | | | | | | | | | 6 |  |  |  |
|  |  | Animal husbandry | | | | | | | | | | | | | | | | | | | | | | | | | | | | | | | | | | 7 |  |  |  |
|  |  | Treatment (Veterinarians/animal handlers) | | | | | | | | | | | | | | | | | | | | | | | | | | | | | | | | | | 8 |  |  |  |
|  |  | Others | | | | | | | | | | | | | | | | | | | | | | | | | | | | | | | | | | 9 |  |  |  |
|  | Consumption | Bushmeat | | | | | | | | | | | | | | | | | | | | | | | | | | | | | | | | | | 1 |  |  |  |
|  |  | Raw meat | | | | | | | | | | | | | | | | | | | | | | | | | | | | | | | | | | 2 |  |  |  |
|  |  | Raw milk | | | | | | | | | | | | | | | | | | | | | | | | | | | | | | | | | | 3 |  |  |  |
|  |  | Others | | | | | | | | | | | | | | | | | | | | | | | | | | | | | | | | | |  |  |  |  |
|  | Environmental exposure | Soil surface | | | | | | | | | | | | | | | | | | | | | | | | | | | | | | | | | | 1 |  |  |  |
|  |  | Water bodies | | | | | | | | | | | | | | | | | | | | | | | | | | | | | | | | | | 2 |  |  |  |
|  |  | Firewood collection | | | | | | | | | | | | | | | | | | | | | | | | | | | | | | | | | | 3 |  |  |  |
|  | Habitat exposure | Habitat proximity to animals | | | | | | | | | | | | | | | | | | | | | | | | | | | | | | | | | | 1 |  |  |  |
|  |  | Domestic animal presence within habitat | | | | | | | | | | | | | | | | | | | | | | | | | | | | | | | | | | 2 |  |  |  |
|  | Recreational exposure | Recreational swimming | | | | | | | | | | | | | | | | | | | | | | | | | | | | | | | | | | 1 |  |  |  |
|  |  | Recreational fishing | | | | | | | | | | | | | | | | | | | | | | | | | | | | | | | | | | 2 |  |  |  |
|  |  | Recreational hunting | | | | | | | | | | | | | | | | | | | | | | | | | | | | | | | | | | 3 |  |  |  |
| **Human-animal contact history (past 3 months)**  **TL**-touching live; **BL**-butchering life; **TD**-touching dead; **BD**-butchering dead; **S**-secretion; **F**-faeces; **T**-type; **AP**-affected part; **S**-self; **C**-clinic; **FR**- forest; **F**-farm; **H**-household; **C**-community; **H**-high; **M**-medium; **L**-low; **O**-other; **R**-rain season; **D**-dry season | | | | | | | | | | | | | | | | | | | | | | | | | | | | | | | | | | | | | | | |
|  | **Wild species** | Direct | | | | Indirect | | Injury | | | Treatment | | | | Season | | | | Location | | | | | | | | | | | Frequency | | | | | |  | | | |
|  |  | **TL** | **BL** | **TD** | **BD** | **S** | **F** | **T** | **AP** | | **S** | **C** | | | **R** | |  | | | **FR** | | **F** | | | **H** | **C** | | **O** | | **H** | | **M** | | | **L** |  | | | |
|  | Monkey |  |  |  |  |  |  |  |  | |  |  | | |  | |  | | |  | |  | | |  |  | |  | |  | |  | | |  | 1 | | |  |
|  | Snakes |  |  |  |  |  |  |  |  | |  |  | | |  | |  | | |  | |  | | |  |  | |  | |  | |  | | |  | 2 | | |  |
|  | Hyena |  |  |  |  |  |  |  |  | |  |  | | |  | |  | | |  | |  | | |  |  | |  | |  | |  | | |  | 3 | | |  |
|  | Squirrel |  |  |  |  |  |  |  |  | |  |  | | |  | |  | | |  | |  | | |  |  | |  | |  | |  | | |  | 4 | | |  |
|  | Bush pigs |  |  |  |  |  |  |  |  | |  |  | | |  | |  | | |  | |  | | |  |  | |  | |  | |  | | |  | 5 | | |  |
|  | Fox |  |  |  |  |  |  |  |  | |  |  | | |  | |  | | |  | |  | | |  |  | |  | |  | |  | | |  | 6 | | |  |
|  | Rodent |  |  |  |  |  |  |  |  | |  |  | | |  | |  | | |  | |  | | |  |  | |  | |  | |  | | |  | 7 | | |  |
|  | Rabbit |  |  |  |  |  |  |  |  | |  |  | | |  | |  | | |  | |  | | |  |  | |  | |  | |  | | |  | 8 | | |  |
|  | Antelope |  |  |  |  |  |  |  |  | |  |  | | |  | |  | | |  | |  | | |  |  | |  | |  | |  | | |  | 9 | | |  |
|  | Scorpion |  |  |  |  |  |  |  |  | |  |  | | |  | |  | | |  | |  | | |  |  | |  | |  | |  | | |  | 10 | | |  |
|  | Monitor lizard |  |  |  |  |  |  |  |  | |  |  | | |  | |  | | |  | |  | | |  |  | |  | |  | |  | | |  | 11 | | |  |
|  | Chameleon |  |  |  |  |  |  |  |  | |  |  | | |  | |  | | |  | |  | | |  |  | |  | |  | |  | | |  | 12 | | |  |
|  | Owl |  |  |  |  |  |  |  |  | |  |  | | |  | |  | | |  | |  | | |  |  | |  | |  | |  | | |  | 13 | | |  |
|  | Bat |  |  |  |  |  |  |  |  | |  |  | | |  | |  | | |  | |  | | |  |  | |  | |  | |  | | |  | 14 | | |  |
|  | Vulture |  |  |  |  |  |  |  |  | |  |  | | |  | |  | | |  | |  | | |  |  | |  | |  | |  | | |  | 15 | | |  |
|  | Hipopotamus |  |  |  |  |  |  |  |  | |  |  | | |  | |  | | |  | |  | | |  |  | |  | |  | |  | | |  |  | | |  |
|  | Other |  |  |  |  |  |  |  |  | |  |  | | |  | |  | | |  | |  | | |  |  | |  | |  | |  | | |  |  | | |  |
|  | **Domestic and livestock species (past 3 months)**  **TL**-touching live; **BL**-butchering life; **TD**-touching dead; **BD**-butchering dead; **S**-secretion; **F**-faeces; **T**-type; **AP**-affected part; **S**-self; **C**-clinic; **FR**- forest; **F**-farm; **H**-household; **C**-community; **H**-high; **M**-medium; **L**-low; **O**-other; **R**-rain season; **D**-dry season | | | | | | | | | | | | | | | | | | | | | | | | | | | | | | | | | | | | | | |
|  |  | **TL** | **BL** | **TD** | **BD** | **S** | **F** | **T** | **AP** | | **ST** | | **C** | **R** | | | |  | | | **FR** | **F** | | **H** | | | **C** | | **O** | | **H** | | **M** | **L** | |  | |  | |
|  | Cat |  |  |  |  |  |  |  |  | |  | |  |  | | | |  | | |  |  | |  | | |  | |  | |  | |  |  | | 1 | |  | |
|  | Dog |  |  |  |  |  |  |  |  | |  | |  |  | | | |  | | |  |  | |  | | |  | |  | |  | |  |  | | 2 | |  | |
|  | Chicken |  |  |  |  |  |  |  |  | |  | |  |  | | | |  | | |  |  | |  | | |  | |  | |  | |  |  | | 3 | |  | |
|  | Duck |  |  |  |  |  |  |  |  | |  | |  |  | | | |  | | |  |  | |  | | |  | |  | |  | |  |  | | 4 | |  | |
|  | Rabbit |  |  |  |  |  |  |  |  | |  | |  |  | | | |  | | |  |  | |  | | |  | |  | |  | |  |  | | 5 | |  | |
|  | Sheep |  |  |  |  |  |  |  |  | |  | |  |  | | | |  | | |  |  | |  | | |  | |  | |  | |  |  | | 6 | |  | |
|  | Goat |  |  |  |  |  |  |  |  | |  | |  |  | | | |  | | |  |  | |  | | |  | |  | |  | |  |  | | 7 | |  | |
|  | Cattle |  |  |  |  |  |  |  |  | |  | |  |  | | | |  | | |  |  | |  | | |  | |  | |  | |  |  | | 8 | |  | |
|  | Horse |  |  |  |  |  |  |  |  | |  | |  |  | | | |  | | |  |  | |  | | |  | |  | |  | |  |  | | 9 | |  | |
|  | Other |  |  |  |  |  |  |  |  | |  | |  |  | | | |  | | |  |  | |  | | |  | |  | |  | |  |  | | 10 | |  | |
| **Animal-animal contact**  *Domestic-wild animal contacts (past 3 months)*  Have you ever shared animal care equipment or vehicles with other animal owners?  Yes_____ No__________, if yes list them___________________________  Have you ever allowed your domestic or livestock animals to roam freely in areas where they may come into contact with wild animals?  Yes_____ No__________, if yes list them___________________________  Have you seen wild animals in the same areas where you keep or graze your domestic or livestock animals?  Yes_____ No__________, if yes list them___________________________  Have you ever observed your domestic or livestock animals interacting with wild animals or coming in contact with wild animals?  Yes_____ No__________, if yes list them___________________________  *Domestic-domestic animal contacts (past 3 months)*  Do your animals have access to shared water sources or feed with other animals?  Yes_____ No__________  Do your animals interact with other animals in the village?  Yes_____ No__________  Do you keep your domestic animals together in the same enclosure or surrounding?  Yes_____ No__________  Have you introduced a new animal to your herd or flock in the in past 3 months?  Yes_____ No__________  Have you noticed any signs of illness or disease in your animals in the past few weeks or months?  Yes_____ No__________  Have you vaccinated or treated your animals against any communicable diseases?  Yes , List them_____________________ No__________  *Human vector contact*  How often have you been bitten by mosquitoes in the past week?  High_____ (everyday) in the past week, Medium________ (3-6 times), Low______ (3 and below times) None (not bitten past week)  In what activities do you typically engage that may increase your risk of coming into contact with mosquitoes?  outdoor work_____ recreation_____, sleeping without mosquito nets_____farming_____hunting____  When do you normally get bitten by mosquitoes?  Morning_____________afternoon_________evening_____________night_______________  Have you experienced any symptoms of a vector-borne disease (e.g., fever, rash, joint pain) in the past 1 month?  _____________________________________________________________________________  How frequently do you use mosquito nets or other measures (e.g. insecticide) to prevent mosquito bites?  High_____ (everyday) in the past week, Medium________ (3-6 times), Low______ (3 and below times) None (not bitten past week)  Have you noticed any changes in your own behaviour or living conditions that may have affected your risk of coming into contact with mosquitoes or other disease vectors (e.g., moving to a new area, changing jobs)?  Yes , ______________No__________, if yes list them_____________ | | | | | | | | | | | | | | | | | | | | | | | | | | | | | | | | | | | | | | | |
| Health | | | | | | | | | | | | | | | | | | | | | | | | | | | | | | | | | | | | | | | |
|  | Diagnosed illness in the past 1 month | List them………... | | | | | | | | | | | | | | | | | | | | | | | | | | | | | | | | | | |  | | |
|  | Symptoms of illness in the past 1 month | List them………. | | | | | | | | | | | | | | | | | | | | | | | | | | | | | | | | | | |  | | |
| Treatment facility in order frequently used | |  | | | | | | | | | 1st | | | | | 2nd | | | | | | | 3rd | | | | | | 4th | | | | | | |  |  | | |
|  |  | Clinic | | | | | | | | |  | | | | |  | | | | | | |  | | | | | |  | | | | | | | 1 |  | | |
|  |  | Hospital | | | | | | | | |  | | | | |  | | | | | | |  | | | | | |  | | | | | | | 2 |  |  |  |
|  |  | Self-medication | | | | | | | | |  | | | | |  | | | | | | |  | | | | | |  | | | | | | | 3 |  |  |  |
|  |  | Traditional | | | | | | | | |  | | | | |  | | | | | | |  | | | | | |  | | | | | | | 4 |  |  |  |
|  | Medical check-up | Yes | | | | | | | | | | | | | | | | | | | | | | | | | | | | | | | | | | 0 |  | | |
|  |  | No | | | | | | | | | | | | | | | | | | | | | | | | | | | | | | | | | | 1 |  |  |  |
|  | Lack fund for treatment in the Past 1 month | Yes | | | | | | | | | | | | | | | | | | | | | | | | | | | | | | | | | | 0 |  | | |
|  |  | No | | | | | | | | | | | | | | | | | | | | | | | | | | | | | | | | | | 1 |  |  |  |
|  | Medication adherence | Yes | | | | | | | | | | | | | | | | | | | | | | | | | | | | | | | | | | 0 |  | | |
|  |  | No | | | | | | | | | | | | | | | | | | | | | | | | | | | | | | | | | | 1 |  |  |  |
|  | Decline hospital because of stigma | Yes | | | | | | | | | | | | | | | | | | | | | | | | | | | | | | | | | | 0 |  | | |
|  |  | No | | | | | | | | | | | | | | | | | | | | | | | | | | | | | | | | | | 1 |  |  |  |
|  | Vaccination history (All time) | List them______________________ | | | | | | | | | | | | | | | | | | | | | | | | | | | | | | | | | |  |  | | |
|  | Do you vaccinate on time | Yes | | | | | | | | | | | | | | | | | | | | | | | | | | | | | | | | | | 0 |  | | |
|  |  | No | | | | | | | | | | | | | | | | | | | | | | | | | | | | | | | | | | 1 |  |  |  |
|  | Do you have to work when ill | Yes | | | | | | | | | | | | | | | | | | | | | | | | | | | | | | | | | | 0 |  | | |
|  |  | No | | | | | | | | | | | | | | | | | | | | | | | | | | | | | | | | | | 1 |  |  |  |
|  | Medical aid last 12 months | Yes | | | | | | | | | | | | | | | | | | | | | | | | | | | | | | | | | | 0 |  | | |
|  |  | No | | | | | | | | | | | | | | | | | | | | | | | | | | | | | | | | | | 1 |  |  |  |
|  | Days of hospital visit  (past 1 month) |  | | | | | | | | | | | | | | | | | | | | | | | | | | | | | | | | | | |  | | |
|  | Death past 12 months | Adult | | | | | | | | | Number | | | | | | | | | | | | | | | | | | | | | | | | | |  | | |
|  |  |  |  |  |  |  |  |  |  |  | Age | | | | | | | | | | | | | | | | | | | | | | | | | |  |  |  |
|  |  |  |  |  |  |  |  |  |  |  | Reason | | | | | | | | | | | | Disease | | | | | | | | | | | | | 1 |  |  |  |
|  |  |  |  |  |  |  |  |  |  |  |  |  |  |  |  |  |  |  |  |  |  |  | Accident | | | | | | | | | | | | | 2 |  |  |  |
|  |  |  |  |  |  |  |  |  |  |  |  |  |  |  |  |  |  |  |  |  |  |  | Unknown | | | | | | | | | | | | | 3 |  |  |  |
|  |  | Children | | | | | | | | | Number | | | | | | | | | | | | | | | | | | | | | | | | | |  |  |  |
|  |  |  |  |  |  |  |  |  |  |  | Age | | | | | | | | | | | | | | | | | | | | | | | | | |  |  |  |
|  |  |  |  |  |  |  |  |  |  |  | Reason | | | | | | | | | | | | Disease | | | | | | | | | | | | | 1 |  |  |  |
|  |  |  |  |  |  |  |  |  |  |  |  |  |  |  |  |  |  |  |  |  |  |  | Accident | | | | | | | | | | | | | 2 |  |  |  |
|  |  |  |  |  |  |  |  |  |  |  |  |  |  |  |  |  |  |  |  |  |  |  | Unknown | | | | | | | | | | | | | 3 |  |  |  |
| Nutrition (past 1 month) | | | | | | | | | | | | | | | | | | | | | | | | | | | | | | | | | | | | | | | |
|  | Daily Meal | Once | | | | | | | | | | | | | | | | | | | | | | | | | | | | | | | | | | 1 |  | | |
|  |  | Twice | | | | | | | | | | | | | | | | | | | | | | | | | | | | | | | | | | 2 |  |  |  |
|  |  | Thrice | | | | | | | | | | | | | | | | | | | | | | | | | | | | | | | | | | 3 |  |  |  |
|  |  | Four times | | | | | | | | | | | | | | | | | | | | | | | | | | | | | | | | | | 4 |  |  |  |
|  |  | Five times | | | | | | | | | | | | | | | | | | | | | | | | | | | | | | | | | | 5 |  |  |  |
|  | Days not been able to satisfy food needs |  | | | | | | | | | | | | | | | | | | | | | | | | | | | | | | | | | | |  | | |
|  | Lacked money for feeding | Yes | | | | | | | | | | | | | | | | | | | | | | | | | | | | | | | | | | 0 |  | | |
|  |  | No | | | | | | | | | | | | | | | | | | | | | | | | | | | | | | | | | | 1 |  |  |  |
|  | Food aid past | Yes | | | | | | | | | | | | | | | | | | | | | | | | | | | | | | | | | | 0 |  | | |
|  |  | No | | | | | | | | | | | | | | | | | | | | | | | | | | | | | | | | | | 1 |  |  |  |
|  | Feeding responsibility: | Men | | | | | | | | | | | | | | | | | | | | | | | | | | | | | | | | | | 1 |  | | |
|  |  | Women | | | | | | | | | | | | | | | | | | | | | | | | | | | | | | | | | | 2 |  |  |  |
|  |  | Both | | | | | | | | | | | | | | | | | | | | | | | | | | | | | | | | | | 3 |  |  |  |
| Agricultural practices and decision making process | | | | | | | | | | | | | | | | | | | | | | | | | | | | | | | | | | | | | | | |
|  | Do you have a farm?  (Past 12 months) | Crop farm | | | | | | | | | Private | | | | | | | | | | | | | | | | | | | | | | | | | 1 |  | | |
|  |  |  |  |  |  |  |  |  |  |  | Private-shared | | | | | | | | | | | | | | | | | | | | | | | | | 2 |  |  |  |
|  |  |  |  |  |  |  |  |  |  |  | Rented | | | | | | | | | | | | | | | | | | | | | | | | | 3 |  |  |  |
|  |  |  |  |  |  |  |  |  |  |  | Rented-shared | | | | | | | | | | | | | | | | | | | | | | | | | 4 |  |  |  |
|  |  | Livestock farm | | | | | | | | | Private | | | | | | | | | | | | | | | | | | | | | | | | | 1 |  |  |  |
|  |  |  |  |  |  |  |  |  |  |  | Private-shared | | | | | | | | | | | | | | | | | | | | | | | | | 2 |  |  |  |
|  |  |  |  |  |  |  |  |  |  |  | Rented | | | | | | | | | | | | | | | | | | | | | | | | | 3 |  |  |  |
|  |  |  |  |  |  |  |  |  |  |  | Rented-shared | | | | | | | | | | | | | | | | | | | | | | | | | 4 |  |  |  |
|  | How many do you own?  (Past 12 months) | Crop farm | | | | | | | | | | | | | | | | | | | | | | | | | | | | | | | | | | |  |  |  |
|  |  | Livestock farm | | | | | | | | | | | | | | | | | | | | | | | | | | | | | | | | | | |  |  |  |
|  | Type of farm practice?  (Past 12 months) | Subsistence | | | | | | | | | | | | | | | | | | | | | | | | | | | | | | | | | | 1 |  |  |  |
|  |  | Commercial | | | | | | | | | | | | | | | | | | | | | | | | | | | | | | | | | | 2 |  |  |  |
|  | Perceived Size of farm?  (Past 12 months) | Crop farm | | | | | | | | | Small < 1 hecter | | | | | | | | | | | | | | | | | | | | | | | | | 1 |  |  |  |
|  |  |  |  |  |  |  |  |  |  |  | Med 1-2 hecter | | | | | | | | | | | | | | | | | | | | | | | | | 2 |  |  |  |
|  |  |  |  |  |  |  |  |  |  |  | Large > 3 hecter | | | | | | | | | | | | | | | | | | | | | | | | | 3 |  |  |  |
|  |  | Livestock farm | | | | | | | | | Small < 1 hecter | | | | | | | | | | | | | | | | | | | | | | | | | 4 |  |  |  |
|  |  |  |  |  |  |  |  |  |  |  | Med 1-2 hecter | | | | | | | | | | | | | | | | | | | | | | | | | 5 |  |  |  |
|  |  |  |  |  |  |  |  |  |  |  | Large > 3 hecter | | | | | | | | | | | | | | | | | | | | | | | | | 6 |  |  |  |
|  | Crops planted on the farm?  (Past 12 months) | **Grains** | | | | | | | | | | | | | | | | | | | | | | | | | | | | | | | | | | |  |  |  |
|  |  | Rice | | | | | | | | | | | | | | | | | | | | | | | | | | | | | | | | | | 1 |  |  |  |
|  |  | Beans | | | | | | | | | | | | | | | | | | | | | | | | | | | | | | | | | | 2 |  |  |  |
|  |  | Maize | | | | | | | | | | | | | | | | | | | | | | | | | | | | | | | | | | 3 |  |  |  |
|  |  | G/nut | | | | | | | | | | | | | | | | | | | | | | | | | | | | | | | | | | 4 |  |  |  |
|  |  | Sorghum | | | | | | | | | | | | | | | | | | | | | | | | | | | | | | | | | | 5 |  |  |  |
|  |  | Guinea corn | | | | | | | | | | | | | | | | | | | | | | | | | | | | | | | | | | 6 |  |  |  |
|  |  | Soya beans | | | | | | | | | | | | | | | | | | | | | | | | | | | | | | | | | | 7 |  |  |  |
|  |  | **Vegetable** | | | | | | | | | | | | | | | | | | | | | | | | | | | | | | | | | | |  |  |  |
|  |  | Okra | | | | | | | | | | | | | | | | | | | | | | | | | | | | | | | | | | 1 |  |  |  |
|  |  | Onion | | | | | | | | | | | | | | | | | | | | | | | | | | | | | | | | | | 2 |  |  |  |
|  |  | Sorrel | | | | | | | | | | | | | | | | | | | | | | | | | | | | | | | | | | 3 |  |  |  |
|  |  | Cucumber | | | | | | | | | | | | | | | | | | | | | | | | | | | | | | | | | | 4 |  |  |  |
|  |  | Eggplant | | | | | | | | | | | | | | | | | | | | | | | | | | | | | | | | | | 5 |  |  |  |
|  |  | Pumpkin | | | | | | | | | | | | | | | | | | | | | | | | | | | | | | | | | | 6 |  |  |  |
|  |  | Pepper | | | | | | | | | | | | | | | | | | | | | | | | | | | | | | | | | | 7 |  |  |  |
|  |  | Spinach | | | | | | | | | | | | | | | | | | | | | | | | | | | | | | | | | | 8 |  |  |  |
|  |  | Lettuce | | | | | | | | | | | | | | | | | | | | | | | | | | | | | | | | | | 9 |  |  |  |
|  |  | Tomato | | | | | | | | | | | | | | | | | | | | | | | | | | | | | | | | | | 10 |  |  |  |
|  |  | Potatoes | | | | | | | | | | | | | | | | | | | | | | | | | | | | | | | | | | 11 |  |  |  |
|  |  | **Fruits** | | | | | | | | | | | | | | | | | | | | | | | | | | | | | | | | | | |  |  |  |
|  |  | Cashew | | | | | | | | | | | | | | | | | | | | | | | | | | | | | | | | | | 1 |  |  |  |
|  |  | Mango | | | | | | | | | | | | | | | | | | | | | | | | | | | | | | | | | | 2 |  |  |  |
|  |  | Sugarcane | | | | | | | | | | | | | | | | | | | | | | | | | | | | | | | | | | 3 |  |  |  |
|  |  | Orange | | | | | | | | | | | | | | | | | | | | | | | | | | | | | | | | | | 4 |  |  |  |
|  |  | Watermelon | | | | | | | | | | | | | | | | | | | | | | | | | | | | | | | | | | 5 |  |  |  |
|  |  | Avocado | | | | | | | | | | | | | | | | | | | | | | | | | | | | | | | | | | 6 |  |  |  |
|  |  | Carrot | | | | | | | | | | | | | | | | | | | | | | | | | | | | | | | | | | 7 |  |  |  |
|  |  | Banana | | | | | | | | | | | | | | | | | | | | | | | | | | | | | | | | | | 8 |  |  |  |
|  | | | | | | | | | | | | | | | | | | | | | | | | | | | | | | | | | | | | | | | |
|  | Animals reared on the farm  (Past 12 months) | Chicken | | | | | | | | | | | | | | | | | | | | | | | | | | | | | | | | | | 1 |  | | |
|  |  | Duck | | | | | | | | | | | | | | | | | | | | | | | | | | | | | | | | | | 2 |  |  |  |
|  |  | Rabbit | | | | | | | | | | | | | | | | | | | | | | | | | | | | | | | | | | 3 |  |  |  |
|  |  | Goat | | | | | | | | | | | | | | | | | | | | | | | | | | | | | | | | | | 4 |  |  |  |
|  |  | Sheep | | | | | | | | | | | | | | | | | | | | | | | | | | | | | | | | | | 5 |  |  |  |
|  |  | Cattle | | | | | | | | | | | | | | | | | | | | | | | | | | | | | | | | | | 6 |  |  |  |
|  |  | Donkey | | | | | | | | | | | | | | | | | | | | | | | | | | | | | | | | | | 7 |  |  |  |
|  |  | Horse | | | | | | | | | | | | | | | | | | | | | | | | | | | | | | | | | | 8 |  |  |  |
|  |  | Others | | | | | | | | | | | | | | | | | | | | | | | | | | | | | | | | | | |  |  |  |
|  | Perceived Proximity of farm to forest | Near > 1 km | | | | | | | | | | | | | | | | | | | | | | | | | | | | | | | | | | 1 |  |  |  |
|  |  | Not so far >1 < 2 km | | | | | | | | | | | | | | | | | | | | | | | | | | | | | | | | | | 2 |  |  |  |
|  |  | Far > 3 km | | | | | | | | | | | | | | | | | | | | | | | | | | | | | | | | | | 3 |  |  |  |
|  | Months of agricultural activities (clearing to harvest) | Jan | | | | | | | | | | | | | | | | | | | | | | | | | | | | | | | | | | 1 |  |  |  |
|  |  | Feb | | | | | | | | | | | | | | | | | | | | | | | | | | | | | | | | | | 2 |  |  |  |
|  |  | March | | | | | | | | | | | | | | | | | | | | | | | | | | | | | | | | | | 3 |  |  |  |
|  |  | April | | | | | | | | | | | | | | | | | | | | | | | | | | | | | | | | | | 4 |  |  |  |
|  |  | May | | | | | | | | | | | | | | | | | | | | | | | | | | | | | | | | | | 5 |  |  |  |
|  |  | June | | | | | | | | | | | | | | | | | | | | | | | | | | | | | | | | | | 6 |  |  |  |
|  |  | July | | | | | | | | | | | | | | | | | | | | | | | | | | | | | | | | | | 7 |  |  |  |
|  |  | August | | | | | | | | | | | | | | | | | | | | | | | | | | | | | | | | | | 8 |  |  |  |
|  |  | September | | | | | | | | | | | | | | | | | | | | | | | | | | | | | | | | | | 9 |  |  |  |
|  |  | October | | | | | | | | | | | | | | | | | | | | | | | | | | | | | | | | | | 10 |  |  |  |
|  |  | November | | | | | | | | | | | | | | | | | | | | | | | | | | | | | | | | | | 11 |  |  |  |
|  |  | December | | | | | | | | | | | | | | | | | | | | | | | | | | | | | | | | | | 12 |  |  |  |
|  | Who decides crops to be planted | Male adult (> 30yrs) | | | | | | | | | | | | | | | | | | | | | | | | | | | | | | | | | | 1 |  |  |  |
|  |  | Female adult (>30yrs) | | | | | | | | | | | | | | | | | | | | | | | | | | | | | | | | | | 2 |  |  |  |
|  |  | Male youth (15-30yrs) | | | | | | | | | | | | | | | | | | | | | | | | | | | | | | | | | | 3 |  |  |  |
|  |  | Female youth (15-30yrs) | | | | | | | | | | | | | | | | | | | | | | | | | | | | | | | | | | 4 |  |  |  |
|  |  | Advice from a project, NGO, or government | | | | | | | | | | | | | | | | | | | | | | | | | | | | | | | | | | 5 |  |  |  |
|  |  | No answer | | | | | | | | | | | | | | | | | | | | | | | | | | | | | | | | | | 6 |  |  |  |
|  | Most important crop grown in the past 12 months? | List them_____________________________________________________ | | | | | | | | | | | | | | | | | | | | | | | | | | | | | | | | | | |  |  |  |
|  | Did you grow intercropped with other plants in the past 12 months? | Grow alone | | | | | | | | | | | | | | | | | | | | | | | | | | | | | | | | | | 1 |  |  |  |
|  |  | Intercropped | | | | | | | | | | | | | | | | | | | | | | | | | | | | | | | | | | 2 |  |  |  |
|  |  | No answer | | | | | | | | | | | | | | | | | | | | | | | | | | | | | | | | | | 3 |  |  |  |
|  | Do you practice shifting cultivation?  (past 12 months) | Yes | | | | | | | | | | | | | | | | | | | | | | | | | | | | | | | | | | 0 |  |  |  |
|  |  | No | | | | | | | | | | | | | | | | | | | | | | | | | | | | | | | | | | 1 |  |  |  |
|  | About how much of your land did you use for growing during the last 12 months? | All or nearly all (90-100%) | | | | | | | | | | | | | | | | | | | | | | | | | | | | | | | | | | 1 |  |  |  |
|  |  | More than half of it (60-90%) | | | | | | | | | | | | | | | | | | | | | | | | | | | | | | | | | | 2 |  |  |  |
|  |  | About half of it (40-60%) | | | | | | | | | | | | | | | | | | | | | | | | | | | | | | | | | | 3 |  |  |  |
|  |  | Less than half of it (10-40%) | | | | | | | | | | | | | | | | | | | | | | | | | | | | | | | | | | 4 |  |  |  |
|  |  | A small amount (1-10%) | | | | | | | | | | | | | | | | | | | | | | | | | | | | | | | | | | 5 |  |  |  |
|  |  | None (0%) | | | | | | | | | | | | | | | | | | | | | | | | | | | | | | | | | | 6 |  |  |  |
|  |  | No answer | | | | | | | | | | | | | | | | | | | | | | | | | | | | | | | | | | 7 |  |  |  |
|  | Pest on farm  (Past 12 months) | Yes  List them………. | | | | | | | | | | | | | | | | | | | | | | | | | | | | | | | | | | 1 |  |  |  |
|  |  | No | | | | | | | | | | | | | | | | | | | | | | | | | | | | | | | | | | 2 |  |  |  |
|  | What did you do with the main harvest during the last 12 months? | Eat or use at home | | | | | | | | | | | | | | | | | | | | | | | | | | | | | | | | | | 1 |  |  |  |
|  |  | Sell | | | | | | | | | | | | | | | | | | | | | | | | | | | | | | | | | | 2 |  |  |  |
|  |  | Feed to livestock | | | | | | | | | | | | | | | | | | | | | | | | | | | | | | | | | | 3 |  |  |  |
|  |  | Keep seed | | | | | | | | | | | | | | | | | | | | | | | | | | | | | | | | | | 4 |  |  |  |
|  |  | Give away or exchange | | | | | | | | | | | | | | | | | | | | | | | | | | | | | | | | | | 5 |  |  |  |
|  |  | There was no harvest | | | | | | | | | | | | | | | | | | | | | | | | | | | | | | | | | | 6 |  |  |  |
|  |  | No answer | | | | | | | | | | | | | | | | | | | | | | | | | | | | | | | | | | 7 |  |  |  |
|  | How much of harvest was sold during the last 12 months? | All or nearly all (90-100%) | | | | | | | | | | | | | | | | | | | | | | | | | | | | | | | | | | 1 |  |  |  |
|  |  | More than half of it (60-90%) | | | | | | | | | | | | | | | | | | | | | | | | | | | | | | | | | | 2 |  |  |  |
|  |  | About half of it (40-60%) | | | | | | | | | | | | | | | | | | | | | | | | | | | | | | | | | | 3 |  |  |  |
|  |  | Less than half of it (10-40%) | | | | | | | | | | | | | | | | | | | | | | | | | | | | | | | | | | 4 |  |  |  |
|  |  | A small amount (1-10%) | | | | | | | | | | | | | | | | | | | | | | | | | | | | | | | | | | 5 |  |  |  |
|  |  | None (0%) | | | | | | | | | | | | | | | | | | | | | | | | | | | | | | | | | | 6 |  |  |  |
|  |  | No answer | | | | | | | | | | | | | | | | | | | | | | | | | | | | | | | | | | 7 |  |  |  |
|  | Use of fertilizer  In the past 12 months | Inorganic | | | | | | | | | NPK | | | | | | | | | | | | | | | | | | | | | | | | | 1 |  |  |  |
|  |  |  |  |  |  |  |  |  |  |  | Urea | | | | | | | | | | | | | | | | | | | | | | | | | 2 |  |  |  |
|  |  | Organic | | | | | | | | | Animal waste | | | | | | | | | | | | | | | | | | | | | | | | | 3 |  |  |  |
|  |  |  |  |  |  |  |  |  |  |  | Green | | | | | | | | | | | | | | | | | | | | | | | | | 4 |  |  |  |
|  |  |  |  |  |  |  |  |  |  |  | Wood ashes | | | | | | | | | | | | | | | | | | | | | | | | | 5 |  |  |  |
|  | Use of pesticide  In the past 12 months | Yes | | | | | | | | | | | | | | | | | | | | | | | | | | | | | | | | | | 0 |  |  |  |
|  |  | No | | | | | | | | | | | | | | | | | | | | | | | | | | | | | | | | | | 1 |  |  |  |
|  | Harvest in the past 12 months | Manual | | | | | | | | | | | | | | | | | | | | | | | | | | | | | | | | | | 0 |  |  |  |
|  |  | Mechanised | | | | | | | | | | | | | | | | | | | | | | | | | | | | | | | | | | 1 |  |  |  |
|  |  | Early harvest | | | | | | | | | Fear of Theft | | | | | | | | | | | | | | | | | | | | | | | | | 1 |  |  |  |
|  |  |  |  |  |  |  |  |  |  |  | Hunger | | | | | | | | | | | | | | | | | | | | | | | | | 2 |  |  |  |
|  |  |  |  |  |  |  |  |  |  |  | Needed Income | | | | | | | | | | | | | | | | | | | | | | | | | 3 |  |  |  |
|  |  |  |  |  |  |  |  |  |  |  | Erratic rainfall or poor weather | | | | | | | | | | | | | | | | | | | | | | | | | 4 |  |  |  |
|  |  |  |  |  |  |  |  |  |  |  | High market price for crop | | | | | | | | | | | | | | | | | | | | | | | | | 5 |  |  |  |
|  |  |  |  |  |  |  |  |  |  |  | Other | | | | | | | | | | | | | | | | | | | | | | | | | 6 |  |  |  |
|  |  |  |  |  |  |  |  |  |  |  | No answer | | | | | | | | | | | | | | | | | | | | | | | | | 7 |  |  |  |
|  |  | Due harvest | | | | | | | | | | | | | | | | | | | | | | | | | | | | | | | | | | 8 |  |  |  |
|  |  | Late harvest | | | | | | | | | | | | | | | | | | | | | | | | | | | | | | | | | | 9 |  |  |  |
|  | Storage of farm produce in the past 12 months | Storage medium | | | | | | | | | Sack | | | | | | | | | | | | | | | | | | | | | | | | | 1 |  |  |  |
|  |  |  |  |  |  |  |  |  |  |  | Drum | | | | | | | | | | | | | | | | | | | | | | | | | 2 |  |  |  |
|  |  |  |  |  |  |  |  |  |  |  | Plastic | | | | | | | | | | | | | | | | | | | | | | | | | 3 |  |  |  |
|  |  |  |  |  |  |  |  |  |  |  | Other | | | | | | | | | | | | | | | | | | | | | | | | | 4 |  |  |  |
|  |  | Storage facility | | | | | | | | | Farmhouse | | | | | | | | | | | | | | | | | | | | | | | | | 1 |  |  |  |
|  |  |  |  |  |  |  |  |  |  |  | Store | | | | | | | | | | | | | | | | | | | | | | | | | 2 |  |  |  |
|  |  |  |  |  |  |  |  |  |  |  | Traditional granary | | | | | | | | | | | | | | | | | | | | | | | | | 3 |  |  |  |
|  |  |  |  |  |  |  |  |  |  |  | Other | | | | | | | | | | | | | | | | | | | | | | | | | 4 |  |  |  |
|  | Irrigation in the past 12 months | Yes | | | | | | | | Manual hauling | | | | | | | | | | | | | | | | | | | | | | | | | | 1 |  |  |  |
|  |  |  |  |  |  |  |  |  |  | Basin dug around plant | | | | | | | | | | | | | | | | | | | | | | | | | | 2 |  |  |  |
|  |  |  |  |  |  |  |  |  |  | Gravity-fed (river diversion) | | | | | | | | | | | | | | | | | | | | | | | | | | 3 |  |  |  |
|  |  |  |  |  |  |  |  |  |  | Sprinkler | | | | | | | | | | | | | | | | | | | | | | | | | | 4 |  |  |  |
|  |  |  |  |  |  |  |  |  |  | Drip | | | | | | | | | | | | | | | | | | | | | | | | | | 5 |  |  |  |
|  |  |  |  |  |  |  |  |  |  | Electric or diesel pump | | | | | | | | | | | | | | | | | | | | | | | | | | 6 |  |  |  |
|  |  |  |  |  |  |  |  |  |  | Other | | | | | | | | | | | | | | | | | | | | | | | | | | 7 |  |  |  |
|  |  |  |  |  |  |  |  |  |  | No answer | | | | | | | | | | | | | | | | | | | | | | | | | | 8 |  |  |  |
|  |  | No | | | | | | | | | | | | | | | | | | | | | | | | | | | | | | | | | | 9 |  |  |  |
|  | Tillage  (Past 12 months) | By hand | | | | | | | | | | | | | | | | | | | | | | | | | | | | | | | | | | 1 |  |  |  |
|  |  | Animal power | | | | | | | | | | | | | | | | | | | | | | | | | | | | | | | | | | 2 |  |  |  |
|  |  | Machine | | | | | | | | | | | | | | | | | | | | | | | | | | | | | | | | | | 3 |  |  |  |
|  |  | No answer | | | | | | | | | | | | | | | | | | | | | | | | | | | | | | | | | | 4 |  |  |  |
|  | Did you expand your farmland in the past 12 months? | Due to draught | | | | | | | | Yes | | | | | | | | | | | | | | | | | | | | | | | | | | 0 |  |  |  |
|  |  |  |  |  |  |  |  |  |  | No | | | | | | | | | | | | | | | | | | | | | | | | | | 1 |  |  |  |
|  |  | Due to low rainfall | | | | | | | | Yes | | | | | | | | | | | | | | | | | | | | | | | | | | 0 |  |  |  |
|  |  |  |  |  |  |  |  |  |  | No | | | | | | | | | | | | | | | | | | | | | | | | | | 1 |  |  |  |
|  |  | Due to low harvest | | | | | | | | Yes | | | | | | | | | | | | | | | | | | | | | | | | | | 0 |  |  |  |
|  |  |  |  |  |  |  |  |  |  | No | | | | | | | | | | | | | | | | | | | | | | | | | | 1 |  |  |  |
|  |  | Due to increase in demand | | | | | | | | Yes | | | | | | | | | | | | | | | | | | | | | | | | | | 0 |  |  |  |
|  |  |  |  |  |  |  |  |  |  | No | | | | | | | | | | | | | | | | | | | | | | | | | | 1 |  |  |  |
|  |  | For profit reasons | | | | | | | | Yes | | | | | | | | | | | | | | | | | | | | | | | | | | 0 |  |  |  |
|  |  |  |  |  |  |  |  |  |  | No | | | | | | | | | | | | | | | | | | | | | | | | | | 1 |  |  |  |
|  |  | Soil fertility | | | | | | | | Yes | | | | | | | | | | | | | | | | | | | | | | | | | | 0 |  |  |  |
|  |  |  |  |  |  |  |  |  |  | No | | | | | | | | | | | | | | | | | | | | | | | | | | 1 |  |  |  |
|  | Did you cut down any tree(s) to expand farmland in the past 12 months? | Yes | | | | | | | | | | | | | | | | | | | | | | | | | | | | | | | | | | 0 |  |  |  |
|  |  | No | | | | | | | | | | | | | | | | | | | | | | | | | | | | | | | | | | 1 |  |  |  |
|  |  |  | | | | | | | | |  | | | | | | | | | | | | | | | | | | | | | | | | | |  | | |
| Preventive attitude to infectious diseases (past 12 months)  Regularly = all the time; Occasionally= not on some days; Never = not at all | | | | | | | | | | | | | | | | | | | | | | | | | | | | | | | | | | | | | | | |
|  | Protective clothing | Regularly | | | | | | | | | | | | | | | | | | | | | | | | | | | | | | | | | | 1 |  | | |
|  |  | Occasionally | | | | | | | | | | | | | | | | | | | | | | | | | | | | | | | | | | 2 |  |  |  |
|  |  | Never | | | | | | | | | | | | | | | | | | | | | | | | | | | | | | | | | | 3 |  |  |  |
|  | Hand gloves | Regularly | | | | | | | | | | | | | | | | | | | | | | | | | | | | | | | | | | 1 |  |  |  |
|  |  | Occasionally | | | | | | | | | | | | | | | | | | | | | | | | | | | | | | | | | | 2 |  |  |  |
|  |  | Never | | | | | | | | | | | | | | | | | | | | | | | | | | | | | | | | | | 3 |  |  |  |
|  | Safety boots | Regularly | | | | | | | | | | | | | | | | | | | | | | | | | | | | | | | | | | 1 |  |  |  |
|  |  | Occasionally | | | | | | | | | | | | | | | | | | | | | | | | | | | | | | | | | | 2 |  |  |  |
|  |  | Never | | | | | | | | | | | | | | | | | | | | | | | | | | | | | | | | | | 3 |  |  |  |
|  | Nose mask | Regularly | | | | | | | | | | | | | | | | | | | | | | | | | | | | | | | | | | 1 |  |  |  |
|  |  | Occasionally | | | | | | | | | | | | | | | | | | | | | | | | | | | | | | | | | | 2 |  |  |  |
|  |  | Never | | | | | | | | | | | | | | | | | | | | | | | | | | | | | | | | | | 3 |  |  |  |
|  | Apron | Regularly | | | | | | | | | | | | | | | | | | | | | | | | | | | | | | | | | | 1 |  |  |  |
|  |  | Occasionally | | | | | | | | | | | | | | | | | | | | | | | | | | | | | | | | | | 2 |  |  |  |
|  |  | Never | | | | | | | | | | | | | | | | | | | | | | | | | | | | | | | | | | 3 |  |  |  |
|  | Working cloth | Regularly | | | | | | | | | | | | | | | | | | | | | | | | | | | | | | | | | | 1 |  |  |  |
|  |  | Occasionally | | | | | | | | | | | | | | | | | | | | | | | | | | | | | | | | | | 2 |  |  |  |
|  |  | Never | | | | | | | | | | | | | | | | | | | | | | | | | | | | | | | | | | 3 |  |  |  |
|  | How often do you wash these cloths | Regularly | | | | | | | | | | | | | | | | | | | | | | | | | | | | | | | | | | 1 |  |  |  |
|  |  | Occasionally | | | | | | | | | | | | | | | | | | | | | | | | | | | | | | | | | | 2 |  |  |  |
|  |  | Never | | | | | | | | | | | | | | | | | | | | | | | | | | | | | | | | | | 3 |  |  |  |
|  | How often do you wash your hands | Regularly | | | | | | | | | | | | | | | | | | | | | | | | | | | | | | | | | | 1 |  | | |
|  |  | Occasionally | | | | | | | | | | | | | | | | | | | | | | | | | | | | | | | | | | 2 |  |  |  |
|  |  | Never | | | | | | | | | | | | | | | | | | | | | | | | | | | | | | | | | | 3 |  |  |  |
| Knowledge and perception of zoonosis | | | | | | | | | | | | | | | | | | | | | | | | | | | | | | | | | | | | | | | |
|  | Can you get disease from animals? | Yes, can you name one………………………... | | | | | | | | | | | | | | | | | | | | | | | | | | | | | | | | | | 0 |  | | |
|  |  | No | | | | | | | | | | | | | | | | | | | | | | | | | | | | | | | | | | 1 |  |  |  |
|  | Can you get disease from the environment? | Yes, can you name one………………………... | | | | | | | | | | | | | | | | | | | | | | | | | | | | | | | | | | 0 |  | | |
|  |  | No | | | | | | | | | | | | | | | | | | | | | | | | | | | | | | | | | | 1 |  |  |  |
|  | Can workers (hunter, butcher, etc.) and consumers get disease from meat? | Yes, can you name them.…………………... | | | | | | | | | | | | | | | | | | | | | | | | | | | | | | | | | | 0 |  | | |
|  |  | No | | | | | | | | | | | | | | | | | | | | | | | | | | | | | | | | | | 1 |  |  |  |
|  | Do you think processing dead animal is dangerous? | Yes, can you name one………………………... | | | | | | | | | | | | | | | | | | | | | | | | | | | | | | | | | | 0 |  | | |
|  |  | No | | | | | | | | | | | | | | | | | | | | | | | | | | | | | | | | | | 1 |  |  |  |
|  | Can you get disease from eating raw or undercooked meat? | Yes, can you name one………………………... | | | | | | | | | | | | | | | | | | | | | | | | | | | | | | | | | | 0 |  | | |
|  |  | No | | | | | | | | | | | | | | | | | | | | | | | | | | | | | | | | | | 1 |  |  |  |
|  |  |  | | | | | | | | | | | | | | | | | | | | | | | | | | | | | | | | | |  |  |  |  |
|  | Any health challenges related to your work? | Yes, can you name them.……………………… | | | | | | | | | | | | | | | | | | | | | | | | | | | | | | | | | | 0 |  | | |
|  |  | No | | | | | | | | | | | | | | | | | | | | | | | | | | | | | | | | | | 1 |  |  |  |
