# Supplementary material for: Human–animal contact to inform zoonotic disease risk across gradients of agricultural land use change in the Central River Region (CRR) of The Gambia (ZooContact): a formative study
Source: Front Public Health. 2024 Sep 10;12:1424007. doi: 10.3389/fpubh.2024.1424007 (PMC11419968; doi:10.3389/fpubh.2024.1424007)
Supplement: Supplementary file 2 [file Table_2.DOCX]

**Participant Consent form**

**PROJECT TITLE:** Human-animal contact to inform zoonotic disease risk across gradients of agricultural land-use change in Central River Region CRR of The Gambia (ZooChange)

**STUDY TEAM:**

……………………

Thank you for considering to be part of this research project. The research team must explain to you what the project is about and provide you with a copy of the participant information sheet before you agree to participate in this study. If you have any questions or need further clarification on the information sheet you have been provided with or the explanations given, please ask the research team before you agree to participate. You will be issued a copy of this consent form to keep and refer to, if need be, at any point in time.

I confirm that I have read and understood the information sheet denoted (v1.1_17032022) for the above-named study. I have had the opportunity to consider the information, ask questions and have these answered satisfactorily.

I understand that my consent is voluntary and that I am free to withdraw this consent at any time without giving any reason and without my legal rights being affected.

I understand that relevant sections of my data collected during the study may be looked at by authorised individuals. I grant permission for these individuals to have access to these records for research purposes.

I agree that my anonymised data may be shared with authorised third parties and/or used for future research purposes.

I understand that data about or from me (the participant) may be shared via a public data repository, published, or shared directly with other researchers, and that I will not be identifiable from this information.

I consent that the information generated from my responses can be shared with other researchers elsewhere on request for further studies, as guided by the data sharing policies of the MRC Unit The Gambia.

**SIGNATURES**

I have read, understood, and agreed to all statements contained in this consent form. My questions/concerns have been answered.

My signature or thumbprint below means that I voluntarily agree to participate in the study. I am aware that I can withdraw from the study at any time I wish without any consequences.

Participant signature or thumbprint Date

Investigator/Interviewer Date
