# Supplementary material for: Human–animal contact to inform zoonotic disease risk across gradients of agricultural land use change in the Central River Region (CRR) of The Gambia (ZooContact): a formative study
Source: Front Public Health. 2024 Sep 10;12:1424007. doi: 10.3389/fpubh.2024.1424007 (PMC11419968; doi:10.3389/fpubh.2024.1424007)
Supplement: Supplementary file 3 [file Table_3.DOCX]

**Participant Information Sheet**

**PROJECT TITLE:** Human-animal contact to inform zoonotic disease risk across gradients of agricultural land-use change in Central River Region CRR of The Gambia (ZooChange)

**INTRODUCTION**

This sheet contains all the details on the project and what you as a participant will be asked to do during your participation. Please read carefully and decide whether you want to participate or not.

The study will use a questionnaire to ask you about your contact with animals, information about yourself and your household, farm, forest activities, and hygiene practice. In addition to this, we will take record of the animals and plants you have within and around your household.

**WHY IS THE STUDY CONDUCTED?**

In this project we aim to understand the type of contact you are having with animals and if that poses a risk to human health or leads to transmission of diseases from animals to humans.

**WHAT WE WILL DO DURING THE RESEARCH PROJECT (METHODS)**

The study involves three main activities.

1) The first aspect of the study will use questionnaires to get some personal information about you and your household, your contact with animals, types of animals within and around your compound, and your farming activities

2) In the second part of the study, we will record animal sounds using small recorders that will be placed on trees within the community. This will help us identify the animals that may be present in and around your village. This will help us to understand further what animals are present and the contacts encountered with these animals. We will place a total of eight (4) recorders around the village.

3)We will randomly sample one animal in your house and test if the animal has any of these diseases (rabies, tuberculosis, yellow fever, Rift Valley Fever, dengue fever, West Nile Virus, Brucellosis, and histoplasmosis) that can be transmitted to you and can affect the health of both you and your animal.

**HOW MANY PEOPLE WILL BE IN THE STUDY?**

- The study will include 12 Villages in The Gambia.
- 828 houses and 792 people in total will be involved.

33 houses in every village will be participating in this project. In every house, one person will be selected randomly and interviewed, and one animal will be sampled.

- Male or females of 15 years and above are people who can participate in this study.

**WHAT AM I BEING ASKED TO DO?**

You will be asked certain questions about yourself, your household and contact with animals, farming activities, health condition, nutrition and hygiene practices. Your responses to the questions will be recorded.

**HOW LONG WILL I BE IN THE STUDY?**

Participants will be in the study for a duration of 30 mins to 1 hour maximum.

**WHAT ARE THE BENEFITS OF BEING IN THE STUDY?**

There will be no direct benefit in cash or kind during the study. However, the information from your response will help health care services, scientish to plan ways to mitigate or prevent zoonotic disease outbreaks.

**WHAT ARE THE RISKS OF BEING IN THE STUDY?**

To our knowledge, for interviews, there is no perceived risk to the participants. Only questions pertaining to the study as attached to this document will be asked and recorded.

**WHAT ARE THE COSTS OF BEING IN THE STUDY?**

You do not need to pay anything to participate in this study.

**CONFIDENTIALITY?**

The information we record from you will be confidential. Answers to the questions we will ask you will be used for this study and may be given to other people who will want to use them for further research. In this case, we will ensure that any information that you provide is handled with adequate privacy. Your name or any information that will identify you will be removed and will not be used by us or shared with anyone.

If a photograph or video of you is required at any point during this project, we will seek your permission before we do so. We will also allow you to preview and give permission for the use of any pictures or recordings we take.

**WILL I BE COMPENSATED FOR PARTICIPATING IN THE STUDY?**

There will be no compensation for participants in this study.

**WHAT IF I AM INJURED?**

This study does not involve collecting any samples from you or taking your body measurements, so we do not expect that you will suffer any harm or injury from participating.

**WHAT ARE MY RIGHTS AS A PARTICIPANT?**

You have the right to participate or not to do so. You can ask any person from the study team questions and decide not to continue to participate for any reason or at any time.

You will be informed of any information from your responses during the course of this study that might affect your health or welfare.

**WHO DO I CONTACT IF I HAVE QUESTIONS, CONCERNS, OR COMPLAINTS?**

You are free to contact any member of the study team at any time. You can also make complaints to any person on the team at any point in time.

**CONTACTS**
