# Supplementary material for: Human–animal contact to inform zoonotic disease risk across gradients of agricultural land use change in the Central River Region (CRR) of The Gambia (ZooContact): a formative study
Source: Front Public Health. 2024 Sep 10;12:1424007. doi: 10.3389/fpubh.2024.1424007 (PMC11419968; doi:10.3389/fpubh.2024.1424007)
Supplement: Supplementary file 5 [file Table_5.DOCX]

The questionnaire data generated the expected information on the frequency and diversity of animal contacts as follows:

Frequency of HAC

The most frequent wild animal contact (Fig 3) was with rodents (15/50), monkeys (8/50), bats and scorpions (7/50) respectively. Domestic animal contact (Fig 4) was frequent with goats (30/50), chickens (25/50), and sheep (23/50).

Diversity of HAC

Study villages showed varied contact diversity. Most frequently reported direct contact (Fig 5), TL: touching a live animal was in Saruja (9/50), BL: butchering a live animal was in Tuba Koto (10/50), TD: touching a dead animal was in Wellingara (7/50), BD: butchering a dead animal was in Wellingara and Tuba Koto (3/50). Most frequently reported indirect contact (Fig 6), S: in contact with secretions or touching contaminated surfaces was in Tuba Koto, F: in contact with faeces or contaminated surfaces was in Boiram.

For hygiene practices regarding infectious diseases, most participants reported never wearing protective clothing (38/50), aprons (35/50), nose masks (42/50), safety books (34/50), and hand gloves (38/50).
